# Biomarkers in Motor Neuron Disease: A State of the Art Review

**DOI:** 10.3389/fneur.2019.00291

**Published:** 2019-04-03

**Authors:** Nick S. Verber, Stephanie R. Shepheard, Matilde Sassani, Harry E. McDonough, Sophie A. Moore, James J. P. Alix, Iain D. Wilkinson, Tom M. Jenkins, Pamela J. Shaw

**Affiliations:** Department of Neuroscience, Sheffield Institute for Translational Neuroscience (SITraN), University of Sheffield, Sheffield, United Kingdom

**Keywords:** biomarker, motor neuron disease (MND), ALS (Amyotrophic lateral sclerosis), neuroimaging, cerebrospinal fluid (CSF), electrophysiology, biofluid

## Abstract

Motor neuron disease can be viewed as an umbrella term describing a heterogeneous group of conditions, all of which are relentlessly progressive and ultimately fatal. The average life expectancy is 2 years, but with a broad range of months to decades. Biomarker research deepens disease understanding through exploration of pathophysiological mechanisms which, in turn, highlights targets for novel therapies. It also allows differentiation of the disease population into sub-groups, which serves two general purposes: (a) provides clinicians with information to better guide their patients in terms of disease progression, and (b) guides clinical trial design so that an intervention may be shown to be effective if population variation is controlled for. Biomarkers also have the potential to provide monitoring during clinical trials to ensure target engagement. This review highlights biomarkers that have emerged from the fields of systemic measurements including biochemistry (blood, cerebrospinal fluid, and urine analysis); imaging and electrophysiology, and gives examples of how a combinatorial approach may yield the best results. We emphasize the importance of systematic sample collection and analysis, and the need to correlate biomarker findings with detailed phenotype and genotype data.

## Introduction

Motor neuron disease (MND), or amyotrophic lateral sclerosis (ALS), is a neurodegenerative and ultimately fatal disease that causes progressive muscle weakness through loss of upper and lower motor neurons (UMN and LMN). Non-motor pathways are also affected and up to 50% of patients have detectable cognitive and behavioral changes (1). ALS can be classified as sporadic (sALS) or familial (fALS). Biomarkers in ALS have been the subject of intense research and discussion over the past 20 years. Sensitive and specific biomarkers have the potential to help clinicians and researchers better understand the disease, improve the design of clinical trials, develop novel therapeutics, and improve patient outcomes. A large body of research exists, although this has led to the provision of only a few validated biomarkers. In part, this reflects a wide variation in methodology, non-standardized analytical techniques, small sample sizes and paucity of longitudinal studies. To validate a biomarker there needs to be recognition of the limitations of the analytical technique by which it is being measured, the analysis must use a standardized operating procedure (SOP), and there must be test-retest reliability, ideally across different centers, to ensure replicability. This review aims to summarize progress in biomarker development in the domains of systemic measures including respiratory function, biochemical analysis of biofluids, electrophysiology and imaging. Table 1 summarizes the biomarkers discussed in the article.

**Table 1:** Summary of biomarkers across modalities.

**Biomarker**	**Modality**	**Key findings**	**Salient characteristics and potential applications**
**BIOMETRICS**			
Body weight		5–10% weight loss from baseline	Indicator of poor prognosis
Respiratory function	Sniff nasal inspiratory pressure (SNIP)	Reduction with disease progression or at presentation in respiratory onset disease	Non-invasive, effort-dependent Used clinically as a marker of respiratory function
	Forced/slow vital capacity (FVC/SVC)		Non-invasive, effort-dependent, limited in bulbar weakness Used clinically as a marker of respiratory function and as criteria for trial entry
	Phrenic nerve conduction study		More invasive and requires operator expertise but passive and objective
**BIOFLUID BIOMARKERS**			
Genetic mutation-linked proteins	CSF	C9orf72 poly(GP) present pre-clinically; stable over time SOD1 protein levels stable over time	Pharmacodynamic potential for clinical trials
	Blood	Level of SOD1 proteins in familial and sporadic disease poly(GP) repeats present in *C9ORF72* disease TDP-43 mislocalized but longitudinal readouts variable	SOD1 used in current clinical trial Planned clinical trial specific to *C9ORF72* mutations Potential as markers for gene-specific disease
DNA methylation	Blood	Conflicting evidence in different cell types Global methylation shows promise	Potential, needs further investigation
Neurodegeneration	CSF	Neurofilament, increased levels of both NfL and pNfH, stable over time	Validated as diagnostic markers. Potential for prognostic and pharmacodynamic monitoring
	Blood	Steady increased NfL over time pNfH levels variable	Potential use of NfL as a diagnostic and prognostic marker
	Urine	p75ECD increased and increases over time	Potential, needs further investigation
Inflammation	CSF	Range of cytokines, chemokines, and immunological proteins up- and downregulated	Potential for diagnostic, prognostic, and disease progression; conflicting evidence currently
	Blood	T regulatory (Treg) cells altered Conflicting results across studies for cytokines, CRP, chitotriosidase	Tregs potential use as prognostic marker, targeted in current phase II trial Other targets need further investigation
Muscle denervation	Blood	Serum creatinine reduction Longitudinal changes in creatine kinase	Serum creatinine potential as prognostic marker Creatine kinase predicts slow vs. fast disease progression in panel in PRO-ACT database
miRNA	*CSF*	Differences in panels of miRNAs in patients Paucity of overlap across studies	Early potential for diagnostic, prognostic and pharmacodynamic; needs further investigation
	Blood	As per CSF	
Metabolism	CSF	Distinctive lipid profile identified through 1H-NMR and mass spectrometry Inconsistencies across studies	Potential for diagnostic and prognostic use Longitudinal studies needed
	Blood	Carbohydrate and lipid metabolism markers contradictory, but larger study promising Glutamate results contradictory in response to treatment Serum albumin reduction	Carbohydrate and lipid metabolism markers associated with disease risk in large 20-year study Glutamine and glutamate need further investigation Serum albumin predicts slow vs. fast disease progression in panel in PRO-ACT database
	Urine	Limited studies on F2-isoprostane (8-iso-PGF2α), Collagen type 4, and lucosylgalactosyl hydroxylysine (glu-gal Hyl)	Potential, needs further investigation
Oxidative stress	CSF	Raised levels of 4HNE, 3-nitrotyrosine NRF-2 pathway markers e.g., glutathione	Needs further investigation
	Blood	^1^Uric acid results contradictory, but larger study promising Ferritin, glutathione, 3-nitrotyrosine, 4HNE increase	Uric acid shows promise as prognostic in PRO-ACT database Other candidates need further investigation
	Urine	8-hydroxy-2′-deoxyguanosine (8-OhdG) increased and increases over time	Potential, needs further investigation
**BIOMETRICS**			
Proteomic approach	CSF	Differential expression profiles identified e.g., cystatin C, chitinases, MCP-1, Subsequent failure of validation of individual markers	Potential as an unbiased investigation of novel markers but inconsistency across studies and validation of findings needed
**IMAGING BIOMARKERS**			
Central nervous system Magnetic Resonance Imaging (MRI) and Magnetic Resonance Spectroscopy	Structural MRI	Focal atrophy Subcortical hyperintensities on T2 weighted, Proton Density weighted, and Fluid-Attenuated Inversion Recovery images Cortical hypointensities on T2-weighted, T2^*^-weighted, and Susceptibility Weighted Images	Employed in clinical practice to exclude mimics Cervical cord atrophy might have potential as a predictive and progression biomarker The potential use of cortical hypointensities as a biomarker is currently being explored
	Diffusion tensor imaging	Fractional Anisotropy reduction Mean Diffusivity elevation	Potential use as a biomarker of is under investigation
	Magnetization transfer imaging	Possible reduction in Magnetization Transfer Imaging ratios	Conflicting evidence
	Functional magnetic resonance imaging	Cortical reorganization	Useful primarily to explore pathogenesis; might provide evidence of target engagement in clinical trials
	Proton magnetic resonance spectroscopy	N-acetylaspartate reduction	N-acetylaspartate has been suggested as a diagnostic and disease progression biomarker and has been employed in a clinical trial
Peripheral nerve MRI	Diffusion tensor imaging	Fractional Anisotropy reduction	Potential use as a biomarker of disease progression
Muscle MRI and MRS	Anatomical imaging	Muscle volume reduction T2 hyperintensities	Potential use as a biomarker of disease progression
	Phosphorus magnetic resonance spectroscopy	Conflicting evidence	Technique's potential as a marker of energy dysmetabolism has not yet been fully explored
Positron emission tomography		Alterations in Fluoro-2-deoxy-2-D-glucose uptake Enhanced microglial activation Inhibitory inter-neuronopathy Alterations of serotoninegic neurotransmission Increased oxidative stress	Potential diagnostic biomarker and use in clinical trials to provide evidence of target engagement
**ELECTROPHYSIOLOGY BIOMARKERS**			
Motor unit number estimation	MUNE	Sensitive to disease progression Identifies pre-clinical LMN loss (MPS method)	Principally limited by operator-dependent variation in recording Newer methods (e.g., MScanFIT) expedite recording and overcome some technical limitations, but require dedicated software and evaluator training Potential for use diagnosis and follow-up Yet to be widely employed clinically
	MUNIX	Multicenter and multi-operator reliability and sensitivity demonstrated Positive influence of evaluator training Superior sensitivity to early disease change vs. conventional methods Identifies pre-clinical LMN loss	Relatively time-efficient and tolerable for patients Dependent upon patient cooperation as derived from muscle contraction Worldwide evaluation in clinical trials Commercially available
Neurophysiological index		Increased distal motor latency and F-wave frequency Decreased CMAP amplitude Sensitive to disease change in 4 weeks, greater rate of decline vs. ALSFRS-R, CMAP amplitude, and FVC	Utilizes standard neurophysiological measures Previously employed in clinical trials Potential to reduce required trial duration Further investigation required
Axonal excitability		Upregulation of persistent Na^+^ conductances Reduction of slow and fast K^+^ channel conductances Change with disease progression	Predictor for poor prognosis Specialist equipment Further investigation required
Electrical impedance myography		Multicentre demonstration of sensitivity to disease progression Applicable to bulbar musculature	Simple technique requiring limited patient cooperation or operator training Potential to reduce required sample size Further investigation into diagnostic utility and technique optimization required
Transcranial magnetic stimulation		Reduced short-interval intracortical inhibition, cortical silent-period duration, and resting motor threshold Increased intracortical facilitation and motor evoked potential Discriminates ALS from mimics	Specialist equipment/software Further multicenter investigation confirming diagnostic utility and evaluating longitudinal potential required

### Diagnostic Biomarkers

**Figure 1:**
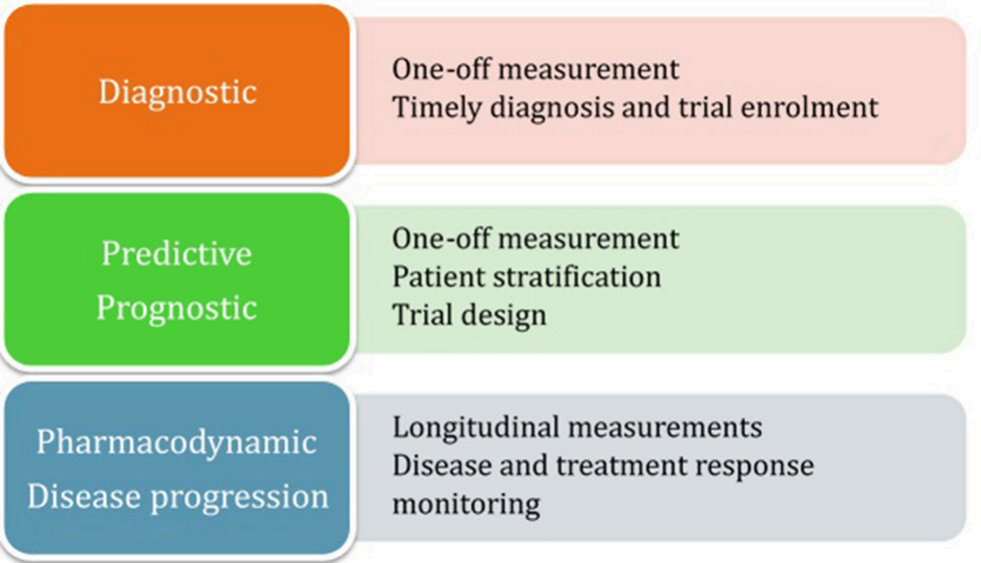
Summary of biomarker categorization.

ALS patients may initially present with subtle signs and symptoms and it has been shown that, on average, there is a 12-month period between symptom onset and neurological diagnosis (2). Current thinking is that there is pathological propagation of the disease through mechanisms such as axonal transmission of misfolded protein e.g., pTDP-43 [associated with diffusion tensor imaging (DTI)], abnormal RNA processing, “prion-like” spread, and cell-cell spread of dipeptide repeat proteins (3–8). It is hoped that by hastening the diagnosis future treatments will limit or halt progression, before patients are established on this progressive pathological course and before they suffer notable weakness and attrition of motor neuron numbers. A valid diagnostic biomarker will help guide the clinical diagnostic process at an early stage when signs are localized and subtle. This would allow for timely treatment and trial enrolment (Figure 1). Diagnosis for enrolment in trials is often based on the El-Escorial criteria which allows for a label of ALS-probable, lab-supported. Currently, this is based on evidence of active and chronic denervation on the electromyogram (EMG), together with the absence of other investigation findings that may suggest another pathological process. Further biomarkers may add to this laboratory support for more accurate enrolment and stratification. Ultimately this stratification may form the basis for a new classification system.

### Prognostic and Predictive Biomarkers

ALS is a heterogeneous condition with variability in site of onset, extra-motor involvement and rate of progression. Typical survival is 2–5 years, but life expectancy can range from several months to over 10 years. This heterogeneity is also seen when patients are investigated at a genetic level, and at post-mortem. It makes sense therefore to design clinical trials with this in mind: a subgroup of patients may be shown to benefit from a novel treatment when statistical analysis is not confounded by population heterogeneity. Additionally, if variability is decreased then sample-size can also be reduced, lowering the time and cost of clinical trials. A good prognostic biomarker will be useful in stratifying patients for better trial design by broadly distinguishing between disease sub-groups. Predictive biomarkers are similar one-off measurements. However, they represent the chance of predicting a response to a particular treatment rather than the prognostic natural course of the disease.

### Pharmacodynamic and Disease Progression Biomarkers

Clinical trial endpoints typically involve measures such as survival and the revised ALS functional rating scale (ALSFRS-R), given that improvement in motor function and survival in a progressive disease are the ultimate outcomes being sought. Such outcomes may need to be monitored for several years before a conclusion can be drawn, which is an expensive process. Pharmacodynamic biomarkers reliably change in response to treatment, and such markers would ensure that an experimental drug is having the desired effect on the pre-clinically identified therapeutic pathway. This could curtail ineffective therapeutic interventions at an early stage. Similarly, disease progression markers represent serial measures that change as the disease worsens, in the absence of treatment. This can provide another objective measure and time-saving approach to randomized control trial design.

## Systemic Prognostic Biomarkers

### Body Weight

An important facet of ALS management entails keeping weight records, prompt insertion of gastrostomy and prescription of nutritional supplements. Malnutrition (defined by a reduction in BMI or a >5% loss in premorbid weight) has a multifactorial adverse effect on life expectancy in ALS, in part due to neurotoxicity (9), and has been shown to give a 7.7-fold increased risk of death across a group of ALS patients at various time-points in the disease course (10). At time of diagnosis, 5% weight loss or more has been shown to be an independent adverse prognostic biomarker for survival (11). Therefore, patient stratification for trial entry, at any point in the disease course, should take into consideration the percentage of weight loss at baseline.

### Respiratory Function

Clinicians rely on patient-reported symptoms of respiratory insufficiency, such as orthopnea, early morning headache, interrupted sleep, daytime somnolence, reduced appetite, and results of respiratory function tests to assess the need for non-invasive ventilation (NIV), which has been shown to improve survival and quality of life in ALS patients (12). Several tests exist and they can be classified according to the time they take, how invasive they are, and whether they require patient volition. Tests such as vital capacity (VC), sniff nasal inspiratory pressure (SNIP), peak cough flow (PCF), maximal static inspiratory and expiratory mouth pressures (MIP and MEP) take a snapshot of respiratory function, but can be confounded by poor technique secondary to non-respiratory muscle weakness and cognitive dysfunction. Overnight sleep studies and transcutaneous carbon dioxide monitoring are passive tests. Tests involving phrenic nerve stimulation—phrenic nerve conduction studies (PNCS) and twitch trans-diaphragmatic pressure (Tw Pdi) are more invasive and complex as they require electrophysiology practitioners, but are objective and non-volitional.

Measures of VC, forced and slow, are widely used due to clinical availability and published validation (13, 14). In a recent study comparing tests as predictive for mortality or NIV usage, Polkey et al. concluded that, despite good sensitivity, decline in vital capacity only occurs 12 months before these endpoints. Furthermore, for prognostic time intervals beyond 3 months, the cut-off value for poor prognosis was >80% predicted, which is the clinically defined normal range, therefore making it an invalid biomarker for trial stratification. A better measure, they argue, would be Tw Pdi or SNIP (15). As Tw Pdi is considered more invasive and complex, SNIP therefore has better potential as a biomarker in clinical practice. This is supported by another study that investigated the ability of respiratory tests to predict the need for NIV over the following 3 month period and found significant reduction in SNIP values in patients going on to require NIV (16). Although 3 months is not long enough for a stratification tool, it strengthens SNIP as a predictive tool. Lending further support to SNIP as a prognostic biomarker, an Italian research team concluded that SNIP measurements at baseline represent an excellent predictor for mortality or tracheostomy within 1 year of follow-up (17).

Sniff nasal testing confers an additional benefit in that it does not rely on the patient being able to form a tight mouth seal around a device, therefore making it better in patients with bulbar weakness (18). It does not completely alleviate the problem however, as upper airway collapse and inability to completely close the mouth also affects SNIP readings to a degree (19). Jenkins et al. also raise concern about using volitional measures for this reason. They concluded from a large prospective study that PNCS to measure diaphragmatic compound muscle action potential (CMAP) has merit as a biomarker as it correlates well with ALSFRS-R, SNIP and FVC, and, after a period of practitioner familiarity, it is as reliable as normal nerve conduction studies and no more difficult to execute (20).

In addition to LMN weakness affecting the respiratory muscles, hypotonic, and weak upper airway muscles contribute to an obstructive picture, and there are central factors contributing to respiratory insufficiency with bulbar, motor, and extra-motor pathways involved. Dysfunctional breathing due to abnormalities in these pathways leads to overnight hypoxia and hypercapnia (21). Clinically, sleep studies are typically reserved for patients who are symptomatic or have fallen below a threshold on screening tests such as VC. They are more cumbersome for patients, and time and resource intensive, which reduces their utility as a biomarker. However, one longitudinal study demonstrated the prognostic value of assessing for obstructive sleep apnea, with mean survival being shorter in patients with a higher apnea/hypopnea index. Interestingly SNIP correlated with this measure (22).

Screening tests for respiratory insufficiency are sensitive tools and each modality has its advantages and disadvantages. As a balance in relation to ease of technique, serial measurements, time, and expertise needed, and predictive power, SNIP stands out as a biomarker that could help in defining prognosis as well as the potential for sensitivity to change from therapeutic interventions. The exception is patients with severe bulbar or cognitive dysfunction and in those patients an electrophysiological modality could be of benefit.

## Cerebrospinal Fluid (CSF) Biomarkers

CSF is a useful biofluid for analysis due to the direct proximity with the brain and spinal cord. It is an ultrafiltrate of plasma [although there are CSF homeostatic mechanisms which, for example, maintain ion concentrations that are different to plasma concentrations (23)]. Thus, protein levels in the CSF are considerably less compared to plasma, making analysis likely to be representative of central nervous system (CNS) activity.

### Neurofilament Proteins

The most promising CSF biomarkers identified to date are neurofilament proteins, a cytoskeletal component of neurons that have been shown to accumulate following axonal damage and degeneration and can be measured in CSF (24, 25). Consisting of three subunits, the two of interest are phosphorylated neurofilament heavy chain (pNfH) and neurofilament light chain (NfL). A substantial body of evidence supports neurofilament levels as a diagnostic element (26–30). Both subunits have been validated in one multi-center study as diagnostic biomarkers (29) and pNfH alone in another (31). These studies address the standardization needed by using carefully designed standard operating procedures (SOPs) for sample collection and processing, and checking consistency of neurofilament levels within patient samples and between centers.

Additional marker utility is still to be validated, although many studies provide supporting evidence. A longitudinal study comparing ALS patients with disease and healthy controls found higher NfL levels in ALS patients and higher levels were associated with worse prognosis (32). Similar results were found in a large cohort study but, when analyzing their longitudinal data, they found that only 67% of ALS patients had higher levels at subsequent time points, with some patients having decreasing values over time. This latter group had a higher baseline value suggesting that a plateau of CSF neurofilament levels is reached once the rate of neuronal death has peaked (33). Another study found that NfL (particularly blood-derived) was fairly stable over time, providing a potential pharmacodynamic monitoring tool, and provided further support for CSF NfL as a prognostic marker for patient stratification (34). The authors also found high correlation between serum and CSF NfL, useful as serial blood tests are easier to obtain than serial CSF samples. Furthermore, there is evidence that CSF NfL correlates to disease subtypes, with those with increased UMN burden (32) or more rapid rates of disease progression, independent of age, showing higher baseline levels (35).

Finally, a meta-analysis correlating pNfH with the ALSFRS-R and disease duration demonstrated a significant negative association (36). Validation efforts would therefore be useful for prognostic, disease progression and pharmacodynamic purposes.

### Tau

The tau protein stabilizes neuronal microtubules. Phosphorylated tangles, with tau as the major constituent, are seen in Alzheimer's disease, and ALS when associated with TDP-FTD. Raised total-tau has been reported in the CSF of ALS patients (37, 38), but no difference was found in another study (39) and there was failure to replicate this quantification in a multi-center, standardized collection analysis (31). Additionally, with no studies of tau showing correlation with disease severity or progression, neurofilament is currently the better marker of neuroaxonal degeneration.

### TAR DNA-Binding protein (TDP-43)

Neuronal and glial inclusions of TDP-43 have been implicated in the pathogenesis of sALS and the linked fronto-temporal dementia (FTD) (40) but not SOD1-ALS (superoxide dismutase-1 mutation) (41). Subsequent studies have found elevated TDP-43 levels in the CSF of ALS patients as compared to healthy and neurological controls with neurodegenerative or neuroinflammatory disease (42–44), and higher in levels in ALS than in FTD (45). However, diagnostic accuracy was not demonstrated and a study by Feneberg et al. suggested that as serum concentrations are 200 times higher than CSF levels, as a biomarker, serum TDP-43 may be more appropriate and with pharmacodynamic utility (46). There is little available evidence for use as a marker of disease progression or prognosis and longitudinal studies are needed.

### Proteomics

Another approach to identifying biomarkers is using liquid or gas chromatography (LC/GC) and mass spectrometry (MS) for proteomic analysis. An advantage is that it is an unbiased approach, yielding peaks for biochemical elements that may not have been previously recognized, and which may indicate a targetable, pathogenic pathway. Any protein identified must then be validated and the pathological pathway identified (47). A recent review outlined the problem with such approaches if they are not standardized: individual studies may find hundreds of proteins that differ between patients and controls, but there is only partial overlap between studies and attempts at replication have tended to fail (48). However, many proteins have been identified using these techniques and are currently undergoing further study.

Using LC-MS, Collins et al. demonstrated that the CSF proteome can be used to identify biomarkers and is relatively stable over time (49). In ALS, raised neurofilament, complement C3 and secretogranin I, and reduced cystatin C were amongst the top differentially expressed proteins identified. Additionally, using a machine learning approach they identified and used four classifier proteins—WD repeat-containing protein 63, amyloid-like protein 1, SPARC-like protein 1, and cell adhesion molecule 3—to differentiate between ALS, healthy controls and other neurological disease (83% sensitivity and 100% specificity).

Low levels of cystatin C in the CSF of ALS patients is well-recognized (50–52), although one study failed to find this difference (53). In a multi-center validation study, no difference between ALS patients and controls was seen (31) and there are conflicting data regarding the correlation with rate of disease progression (50, 51). The level of cystatin C has however been shown to correlate with survival time in limb-onset ALS (51) which lends further weight to the argument for careful clinical phenotyping and the need for longitudinal studies.

Other biomarkers analyzed in this six-center analysis (31) were monocyte chemoattractant protein-1, progranulin, amyloid precursor protein and S100B. Of these, none demonstrated consistent change and some yielded conflicting results across the centers.

Chitotriosidase (CHIT1) was identified using a proteomic approach and levels were found to be significantly higher in ALS patients compared to controls (54, 55). A subsequent study using ELISA confirmed this and also found high expression in comparison to other neurodegenerative conditions, and that levels were correlated with progression rate and inversely correlated with disease duration (56). Immunohistochemistry (IHC) was then performed on post-mortem CNS tissue from ALS patients demonstrating CHIT-positive activated microglia and macrophages in the corticospinal tracts. The authors therefore tentatively concluded that CHIT may have a role as a diagnostic and prognostic marker. This is supported by a recent LC-MS longitudinal study which demonstrated CHIT1 and other chitinases, CHI3L1 and CHI3L2, correlate with disease progression and indeed pNfH levels (57).

Levels of glutamate receptor 4 (GRIA4) expression in the CSF were found to be increased in ALS patients and to negatively correlate with disease severity, suggesting an early over-expression. This fits with glutamate excitotoxicity as a factor in neuronal damage and suggests that anti-glutamate therapy, like riluzole, may be more effective earlier in the disease course (55).

### Metabolomics

Like proteomics, an unbiased search can be done by performing LC/MS or proton-nuclear magnetic resonance (^1^H-NMR) on biofluids to identify metabolites that differ in quantity in ALS. One such ^1^H-NMR study demonstrated lower CSF levels of acetate and increased levels of pyruvate and ascorbate (an antioxidant and linked with glutamate-mediated excitotoxicity) when comparing the ALS group with non-neurodegenerative disease controls. Subsequent modeling using the 17 identified metabolites achieved a discrimination rate between ALS and controls of 81.6% (58). A subsequent study from the same group increased the validity of CSF metabolomic ^1^H-NMR spectroscopy as a means to discriminate, by testing their metabolite model on a validation cohort, achieving a sensitivity of 78.9% and specificity of 76.5% (59).

Another mass spectrometry approach investigated the CSF lipid profile of ALS patients (60). As discussed in the blood biomarker section, high lipid levels seem to confer survival benefit and, as the authors of this study explain, the brain composition is rich in lipids with many neuronal and systemic biological processes dependent on lipid homeostasis. They found that there was a distinct ALS lipidomic profile and, based on the baseline CSF analysis, they could provide a predictive model with 71% accuracy for disease progression thus providing a potential diagnostic and prognostic biomarker.

The review by Blasco et al. describes in more detail the large number of metabolites discovered and also the inconsistencies across the body of reported research (61). Longitudinal metabolomic studies with analysis of clinical data are scarcer, although one plasma analysis found that some metabolites did correlate with disease progression (62), and another demonstrated a distinctive plasma profile for patients with LMN disease, albeit only with a small sample size (63). There is promise and further work with pre-analytical and analytical SOPs is indicated.

### Oxidative Stress Biomarkers

Oxidative stress is associated with ALS pathogenesis (64–66), and has potential for novel therapies, as supported by the Japanese and American FDA approval of the free radical scavenger edaravone in recent years. In health, superoxide dismutase 1 has an antioxidant role in converting superoxide free radicals into oxygen and hydrogen peroxide. SOD1-mutations are implicated in a proportion of sporadic and fALS cases through toxic gain of function (67). Misfolded SOD1 can be measured in the CSF; it has been demonstrated that there is no significant difference between SOD1 ALS patients and non-SOD1 patients and between all ALS patients and neurological controls (68, 69). The utility of measuring SOD1 protein levels in CSF is as a pharmacodynamic biomarker, as levels are stable in individual patients over time (69, 70) and antisense oligonucleotide (ASO) SOD1-lowering therapy is effective in rats (69). A phase I/II clinical trial is underway to determine whether ASO-therapy gives the same results in humans (NCT02623699). Furthermore, SOD1 ALS can be sub-classified based upon the specific mutation. This provides useful prognostic information for trial design: for example SOD1 A4V missense, the most common SOD1 disease-causing mutation in the United States, has a significantly worse prognosis compared to other mutations (71).

Other oxidative biomarkers that have been identified as raised in ALS patients are 8-oxodeoxyguanosine and 15-F(2t)-isoprostane in urine (72), 8-hydroxy-2′-deoxyguanosine (8OH2′dG) and 3-nitrotyrosine in CSF (73, 74), and 4-hydroxy-2,3-nonenal in serum and CSF (75). However, none are as yet validated for use in clinical trials.

The nuclear erythroid 2-related factor 2-antioxidant response element (Nrf2-ARE) is an important signaling pathway, shown to reduce oxidative stress and inflammation (76). By measuring markers of oxidative stress, it can be shown that novel therapeutics are having the desired preclinical and clinical effect on this pathway. For example, compound screening identified S[+]-apomorphine as an *in-vivo* inducer of Nrf2 in an ALS mouse model by measuring Nrf2 target genes, and as an attenuator of oxidative stress in patient fibroblasts (77). This therefore supports further exploration of Nrf2 activators, like S[+]-apomorphine, with measurable pharmacodynamic biomarkers.

Upregulated by Nrf2 activation, glutathione is another useful marker of oxidative stress, as it acts as a buffer for reactive oxygen species. Reduced serum levels have been shown when comparing ALS patients and controls (78). Measurable by *in-vivo* ^1^H-MRS, this and other metabolites are discussed further in the imaging section.

As a more general measure of the oxidative system, one study showed that ALS patients had reduced antioxidant capacity with increased advanced oxidation protein products, although interestingly bulbar-onset patients had a protein composition similar to controls (79). Another study demonstrated a higher CSF oxidation-reduction potential (ORP) in ALS patients, and a negative correlation with ALSFRS-R in spinal-onset patients, leading the authors to conclude that it may be a marker of disease progression (80). However, their case-control groups were ALS and non-neurodegenerative neurological controls and a more varied control group encompassing all neurological disease may lend further weight to their preliminary findings.

### Biomarkers of Neuroinflammation

As well as measurable changes in antioxidants, immune and inflammatory mediators have a complex role in the pathophysiology of ALS. Whilst initial activation of microglia and astrocytes may be neuroprotective, a state of chronic activation tips the balance toward neurotoxicity, with up- and down-regulation of a wide variety of humoral and cellular factors (81). Mitchell et al. performed a multiplex ELISA to identify potential biomarker candidates in the CSF of ALS patients. They reported that the 5 cytokines with the greatest difference between ALS and controls were IL-10, IL-6, GM-CSF, IL-2, and IL-15 and when combined, gave a differentiation accuracy of 89% (82). Other differentiating factors that have been identified are CHIT-1 and C3, as discussed earlier, IL-17, bFGF, VEGF, MIP-1b, MIP-1α, MCP-1β, and IFN-γ (83), and follistatin, IL-1α, and kallikrein-5 (84).

Prediction of disease duration has also been proposed through multiplex analysis and immunoassays, with IL-9, IL-5, and IL-12 proving negative predictors and MIP-1β and G-CSF positive predictors (85). IFN-y has been shown to correlate with disease progression (83, 86), and bFGF, VEGF, and MIP-1α have been shown to correlate with longer disease duration (83) further demonstrating the homeostatic attempt of the immune system. This immune profiling provides promise for sub-typing ALS patients and combining identification of pathophysiological factors with discovery of potential therapeutic targets.

### C9ORF72

The hexanucleotide repeat expansion associated with C9ORF72 disease causes accumulation of RNA foci and undergoes non-ATG (RAN) translation, forming C9RAN dipeptides (DPR). Toxicity is thought to be in part due to sequestration of RNA binding proteins (87). Like misfolded SOD1 protein, these DPRs are measurable in CSF (88). A cross-sectional study showed one of these, poly(GP), is detectable in the CSF of C9ORF72 ALS and FTD patients but not controls, and that levels are increased in patients pre-clinically (89). This concept was further explored longitudinally to show that DPR levels are stable over time, supporting their use as a pharmacodynamic biomarker (90). This latter study also demonstrated that poly(GP) levels are reduced with the use of ASOs in C9orf72 cell and mouse models. This provides promising proof-of-concept that a targeted approach to these RNA repeats can mitigate an important pathological process in this disease subtype; especially important for asymptomatic carriers. Indeed, a clinical trial is planned using anti-sense oligonucleotides to lower DPRs in human ALS patients with C9ORF72 mutations.

### MicroRNAs (miRNAs)

Short, non-coding RNAs regulate gene expression by binding to mRNA, thereby reducing translation and promoting mRNA degradation. Specific miRNAs have been associated with neuronal cell identity, synaptic function and glial regulation, and neuroinflammation in ALS (91). Interestingly, miRNA biogenesis is linked to TDP-43 which, as described above, is a pathological hallmark of ALS. TDP-43 binding miRNAs are dysregulated in the CSF and serum of sALS patients (92). Several studies have demonstrated other specific miRNA changes in ALS CSF. For example, upregulation of miR-338-3p (93), and miR181a-5p and downregulation of miR21-5p and miR15b-5p (94). This latter study demonstrated a sensitivity of 90% and specificity of 87% when miRNA ratios were used to differentiate between ALS and healthy controls. Early potential for prognostic or pharmacodynamic biomarker properties can be seen in a murine model which identified CSF miR-218 as correlating with motor-neuron loss and also responsiveness to therapy.

Due to discrepancy between methods and the specific miRNAs identified, further validation efforts are required; a recent study attempted to do this through optimizing RNA extraction and small RNA sequencing (91). Similarly, studying larger, longitudinal cohorts, will hopefully allow correlation of potential miRNA biomarkers with clinical phenotype.

As mentioned above, identification of SOD1 and C9ORF72 mutations is used for ASO trial enrolment, and the respective protein levels as pharmacodynamic biomarkers. In terms of prognosis, certain mutations have been found to infer a different disease course. As examples, C9ORF72 carriers have a higher incidence of fronto-temporal dementia, the specific A4V SOD1 mutation carries a poor prognosis (95), and certain UNC13A single nucleotide variants have been associated with shorter survival and others with longer survival (96). However, data are conflicting and the clinical significance of most mutations is unclear, lending support to larger phenotype-genotype studies. These should be systematic, including patients with seemingly sporadic disease, to accurately reflect the burden of genetic mutation in the population. Interested readers are directed to the Project Mine Project (www.projectmine.com) and the recent review of Al Chalabi et al. on the topic (97).

## Blood Biomarkers

Blood based biomarkers are a useful medium between central and peripheral damage in ALS. While some markers show a correlation with CSF markers, as transfer occurs between CSF and blood, other candidate markers arise from peripheral effects of ALS such as muscle denervation.

### C9ORF72, SOD1, and TDP-43

As introduced above, downstream protein readouts linked to genetic mutations have been explored recently in response to current and planned clinical trials specific to *SOD1* and *C9ORF72* mutations. Although most studies have primary outcomes in CSF (89, 90, 98), SOD1 was reduced in leukocytes (99) but not erythrocytes (98, 99) in response to pyrimethamine treatment in *SOD1* positive disease, and poly(GP) repeats were detected in peripheral blood mononuclear cell lysates in *C9ORF72* positive disease, although levels were not compared to those in CSF (90).

In addition to mutation-specific disease, proteins linked to genetic mutations have been studied more broadly in sALS. For example, overall SOD1 levels are reported to be increased in leukocytes (100). The story for TDP-43 remains unclear; it is mislocalized to cytoplasmic fractions of circulating PBMCs in ALS cases (101), and although total TDP-43 level did not discriminate from controls in these cells (101, 102), increasing levels correlated with disease burden longitudinally (102). In plasma, total TDP-43 is increased in ALS, but longitudinal changes were variable between subjects (103) and in serum, TDP-43 levels were unchanged between disease states, with authors suggesting CSF TDP-43 is blood derived and not useful for ALS diagnosis (46).

### DNA Methylation

DNA methylation, as a readout of epigenetic influence, has gained interest in the last decade. Issues surround DNA methylation levels being influenced by variability between cell types and by immune factors, thus confounding methylation as a specific marker for disease phenotype (104). However, increased methylation of different components has been reported widely in ALS. Increased global DNA methylation been detected in ALS blood in some studies (105, 106), but not in a smaller study of two *SOD1* and two *TARDBP* carriers (107). In *C9ORF72* linked disease, *C9ORF72* itself (108, 109) or its promoter (110, 111) are hypermethylated, with *C9ORF72* hypermethylation showing correlation with G4C2 repeat size (109, 111) and promoter hypermethylation linked to reduced RNA foci and dipeptide repeat protein aggregates in the brain (112). Additionally, an increase in DNA methylation age was associated with disease duration in *C9ORF72* linked disease, with every 5-year increase in DNA methylation age correlating to age of onset 3.2 years earlier, and shorter disease length of 1.5 years. This finding fits with sporadic disease, where increased DNA methylation age was detected in four of five ALS-diagnosed monozygotic twins. In this study, although methylation patterns were most similar between twins, the changes in common across all with ALS implicated glutamate metabolism and the Golgi apparatus (113). Similarly in *SOD1-*linked disease, those with not-fully penetrant SOD1 mutations showed increased DNA methylation in comparison to asymptomatic/pauci-symptomatic individuals, and levels showed a positive correlation with disease duration (106).

### Neurofilament Proteins

NfL levels in serum and CSF have been shown to be highly correlated (34). Blood NfL levels were shown to be significantly higher in ALS patients than healthy controls, and a high initial NfL level was a strong independent predictor of survival. However, levels remain steady over time (34) with high levels in early and later stage disease showing no correlation to El Escorial diagnostic categories (114). Hence, NfL appears to have utility as a diagnostic and prognostic marker, rather than a marker of disease progression.

pNfH has also been studied in blood, and correlates with CSF levels (115, 116). In a meta-analysis of two papers, the blood concentration of pNfH was non-significantly higher in ALS (36). One study showed an association between higher plasma pNfH concentrations and a faster disease progression, but this was only significant at 4 months of follow-up (117). Similarly, higher plasma and serum pNfH was associated with increased mortality over the 12 month follow-up period. The reliability of these results is limited by the small sample size and short follow-up period. A longitudinal study did not show a predictable trajectory of plasma NFH over time: levels increased, decreased, or remained steady as disease progressed (34). While a subgroup with fast progressing disease tended to start with higher pNfH levels which decreased over time, the rate of change could not be used to predict disease progression. Another study showed a tendency for pNfH levels to rise and then fall, but there was substantial variability between subjects (118).

### Inflammatory Markers

Various blood markers of immune activity have been studied. One study measured levels of multiple different immune cells and surface markers in order to generate immune phenotypes for familial and sporadic ALS patients (119). They found that ALS patients had increased immune activity, and could be grouped into two distinct immune profiles. Profile 1 patients were reasonably similar to healthy volunteers, but Profile 2 patients had elevated levels of total leukocytes and mononuclear cells, as well as CD3+, CD4+, CD8+, CD4+CD28+, CD3+CD56+ T-cells, and CD8+CD45RA+ naïve T cells. Profile 2 was associated with younger age, familial ALS and significantly increased survival (a median of 344 weeks, vs. 184 weeks for Profile 1). Within profiles, different leukocyte phenotypes were found to influence survival; for example, Profile 1 patients with higher levels of PD-1+ CD4 T cells survived longer, whereas Profile 2 patients with more CD3+CD56+ T cells survived longer, but neither association held true in the other group. It is unclear whether the altered immune profile in ALS is related to the pathophysiology of the disease or a response to disease activity. There was no longitudinal sampling in this study, so it is unclear how the profiles may change over time, but this study shows they are likely useful for prognosis. Another study found that levels of leukocytes, monocytes and NK cells were increased in ALS patients, and that they increased over time. An increase in total leukocytes and neutrophils, and a decrease in CD4 T cells, were correlated with a decrease in ALSFRS-R (120).

T-regulatory cells (Treg) represent a promising biomarker candidate and a possible therapeutic target. These cells suppress various components of the immune response, including cytokine production and T lymphocyte proliferation. One study found that levels of CD4+CD25High Tregs were reduced in patients with ALS, and that the number of Tregs was inversely correlated with rate of disease progression (121). Another study found that the Tregs from ALS patients had reduced ability to suppress activity of T responder lymphocytes, and that Treg dysfunction was correlated with the rate of disease progression (122). These results support the use of Tregs as a prognostic biomarker. In the latter study, disease burden as measured by the Appel ALS score (AALS) at the time of venepuncture was correlated with Treg dysfunction, which implies a decrease in function over time. However, a longitudinal study is needed to confirm this.

Blood levels of cytokines have been studied widely, including tumor necrosis factor-α (TNF-α) (123–125) interleukin-1β (IL-1β), IL-2, IL-4, IL-5, IL-6, IL-8, IL-10, IL-12p70, and IL-13 which were reported to be increased, while interferon–γ (IFN-γ) was decreased in ALS patients in cross-sectional studies. However, cytokine levels did not change over the course of disease (123). In serum, IL-1β (78), IL-6 (78, 83, 126) IL-8 (60, 78), and IFN-γ (83, 86, 127) are also reported to be increased, whereas serum IL-5 levels are decreased. Serum IL-2 and IL-10 results have been less conclusive (78, 83). A recent meta-analysis (128) combining serum and plasma measurements from 25 studies found TNF-α, TNF-receptor 1, IL-6, IL-1β, IL-8, and VEGF were significantly elevated in ALS, but of note is that results for IL-1β, IL-6, and VEGF may have been skewed by one study. Products of complement activation are also increased in ALS patient blood samples; specifically C3b-alpha-chain in serum (129), and C5a (130, 131) and C5b-9 (131) in plasma, along with a wide range of complement factors in another plasma study (130).

Other inflammatory markers have shown varying results, such as C reactive protein (CRP), which showed no differences in plasma (132) or whole blood (60) at baseline. In serum, CRP was increased in ALS and did not associate with ALS risk or survival in one study (133), but correlated with ALSFRS-R and survival in another (134). Similarly, chitotriosidase, expressed by active tissue macrophages, was increased in dried blood spots of ALS patients compared to healthy individuals, and was higher in those with rapidly progressing disease (135). However, Steinacker et al. (56) found no change in chitotriosidase serum levels in ALS compared to controls in the same study in which CSF levels correlated with disease progression and severity.

## Muscle Denervation Biomarkers

Lower serum creatinine in ALS has been reported, and although some studies have found levels differing by onset site (136) or gender (137) the majority link levels to prognosis (136–140). A recent analysis of trial data from over 1,200 people with ALS found strong longitudinal correlations between serum creatinine and ALSFRS-R score, muscle strength, and overall mortality, indicating that using serum creatinine in trials over 18 months in length would allow a reduction in sample size by 21.5% (141). Lending further support to this pathway as a useful biomarker of muscle denervation, serum creatine kinase (CK) is increased in plasma (132), and serum (140, 142) and correlates with survival in some studies (140, 142). This discrepancy may be attributed to differing rates of disease progression. Modeling of the PRO-ACT database showed those with slow disease progression had stable or slowly declining creatine kinase, whereas people with rapidly declining disease had quickly declining levels. Indeed, along with decreases in weight, alkaline phosphatase, and albumin, creatine kinase decline was able to predict slow vs. fast disease progression (143).

### microRNA (miRNA)

Whole blood (93, 144), serum (145–149), and plasma (150, 151) sourced microRNAs have been studied as possible biomarkers, due to their role in regulating gene expression. In whole blood, six downregulated miRNAs and one upregulated miRNA were identified (144) and a later study confirmed upregulation of miR-338-3p in leukocytes and serum (as well as in CSF and spinal cord) (93). A plasma based study (151) found increased levels of hsa-miR-4649-5p and decreased levels of hsa-miR-4299 in ALS patients vs. controls, but found no significant trend over time. Similarly, a second plasma study identified steady upregulation of two different miRNAs longitudinally (150), one of which, miR-206, is also increased in serum (147).

Serum miR-206 was also increased in a study which reported an increase in miR-143-3p and decrease in miR-374b-5p compared to controls (148). Additionally, this longitudinal study reported that miR-206 levels remained steady, while miR-143-3p levels increased and miR-374b-5p levels decreased over time, and that riluzole had no effect on miRNA levels. Further studies identified different panels of miRNAs differentially expressed in ALS serum compared to controls (146, 149) and also to neurological disease controls (149), noting longitudinal changes in separate sets of miRNAs (149) and higher variability across sporadic disease (146) compared to familial cases. Interestingly, one study identified 30 downregulated miRNAs in ALS, 22 of which were also downregulated in presymptomatic ALS mutation carriers, with some showing a greater degree of downregulation after disease onset (145). MicroRNAs seem to have promise as biomarkers, but there is a lack of overlap in microRNAs identified across different study groups, and to date, little longitudinal evidence reported.

## Metabolic Biomarkers

Markers of carbohydrate and lipid metabolism have been studied extensively, with contradictory results [reviewed in (152)] although dysregulation of these processes is clear. A large 20-year study in Sweden showed lower levels of serum glucose and higher levels of low-density lipoprotein cholesterol (LDL-C), apolipoprotein B (apoB), and apoB/apoA-I ratio during the 20 years before diagnosis, and increasing levels of LDL-C, high-density lipoprotein cholesterol (HDL-C), apoB and apoA-I in the 10 years before diagnosis, in 623 ALS patients. As such an increased risk of ALS was observed with increasing serum LDL-C, apoB, and apoB/apoA-I ratio, and high LDL-C/HDL-C and high apoB/apoA-I ratios, whereas high serum glucose was associated with lower ALS incidence (152).

A decrease in glutamine (153), and an increase in its metabolite glutamate (154), the principal excitatory neurotransmitter in the CNS have been identified in ALS plasma, with increased glutamate levels seen in males, those with spinal onset, and correlated with longer disease duration (155). Interestingly, Riluzole treatment had no effect on plasma glutamate (156) but decreased serum glutamate in another study (157) suggesting usefulness of this measure in response to therapies in serum.

A large 2014 study of 638 ALS patients showed the utility of serum albumin at diagnosis as a biomarker of survival, with levels decreased in ALS, better survival seen with increasing levels, and that albumin levels correlated with markers of inflammatory state (137). A more recent study of 42 ALS patients and 18 healthy controls also showed a decrease in plasma derived serum albumin in ALS regardless of cognitive impairment, but could not detect disease severity or survival time using albumin at one time-point alone (130). Most convincingly, longitudinal modeling of ALS from the PRO-ACT database (143) showed that albumin decline, was one of four factors able to predict disease progression rate.

### Proteomics

While many groups have performed mass spectrometry analyses in blood (102, 154, 158–161), there is not often an overlap in the specific proteins identified and those identified require validation. However, pathways known to be dysregulated in ALS are implicated. For example, the largest study of 172 ALS patients and 50 healthy controls (154) identified a panel of 32 differentially expressed proteins, showing dysregulation of carbohydrate and lipid metabolism, mitochondrial function, and creatinine. A recent study in 42 ALS patients and 18 healthy controls showed downregulation of lipid/cholesterol, and coagulation pathways, inhibition of NO and ROS production in macrophages, and increases in acute phase response and the complement system (130).

### Oxidative Stress Biomarkers

An increase in ferritin, suggesting iron misregulation which promotes oxidative stress, is present in plasma (132) and serum of ALS patients (85, 136, 162), with higher levels associated with poorer survival in some studies (123, 136, 162), but not all (85).

While excess uric acid is harmful, it is also a powerful antioxidant and so could be useful to combat the oxidative stress seen in ALS. In cross sectional studies, serum levels are decreased in comparison to healthy controls (163–166). Higher serum uric acid levels correlated with a moderately decreased risk of the future development of ALS (167), but its link to increased survival is less clear, showing positive results in one study (164), only for men (168), or not at all (165). However, a recent study of the PRO-ACT database including 1,736 ALS cases showed an 11% reduction in risk of death for every 1 mg/dl increase in serum uric acid (169). Uric acid levels have also shown promise in plasma, identifying ALS from neurological disease mimics with high sensitivity as part of a 32 metabolic panel biomarker set although levels were no different between groups alone (154).

## Urine Biomarkers

The search for urinary biomarkers in ALS include small cross-sectional studies, often with contradictory results, such as the usefulness of urinary trace elements (170–172). Those showing promise include the oxidative stress marker 8-hydroxy deoxyguanosine (8-OHdG) a product of nuclear and mitochondrial DNA oxidation which was increased in ALS in cross-sectional studies (72, 74) and increased longitudinally over 9 months in ALS patients (2.9 ng/mg creatinine/year) but not in disease controls (74). F2-isoprostane (8-iso-PGF2α) is also increased in ALS patient urine (72), but the existence of an inflammation-induced pathway for F2-isoprostane generation in addition to lipid peroxidation (173) needs to be considered when interpreting results.

Collagen type 4 (174) and collagen metabolite glucosylgalactosyl hydroxylysine (glu-gal Hyl) (175) levels were decreased in people with ALS as compared to neurological disease controls and healthy individuals, levels were lower in people with longer duration of ALS symptoms in cross-sectional analysis, and correlated with decreased levels in skin (collagen type 4), but did not correlate with muscle power rating (174, 175).

More recently, an increase in the extracellular domain of neurotrophin receptor p75 (p75ECD) was reported in ALS patient urine (176–178), which increases longitudinally as disease progresses (2.3 ng/mg creatinine/year), and provides prognostic potential advantages over clinical parameters of disease onset and change in ALSFRS-R alone (178). These findings suggest that urinary p75ECD has potential for use as a prognostic and pharmacodynamic biomarker.

## Imaging Biomarkers

### Magnetic Resonance Imaging (MRI)

Magnetic resonance imaging (MRI) is an attractive candidate as a biomarker tool as it is non-invasive, relatively inexpensive, and does not involve ionizing radiation. The multi-modal nature of MR lends itself to the study of various anatomical and pathological changes and processes *in vivo* (179). There is a large body of published work in the context of ALS, predominantly focused on the brain, with fewer studies relating to the spinal cord, muscle, and peripheral nerve.

## Central Nervous System

### Conventional Anatomical Magnetic Resonance Imaging (MRI)

**Figure 2:**
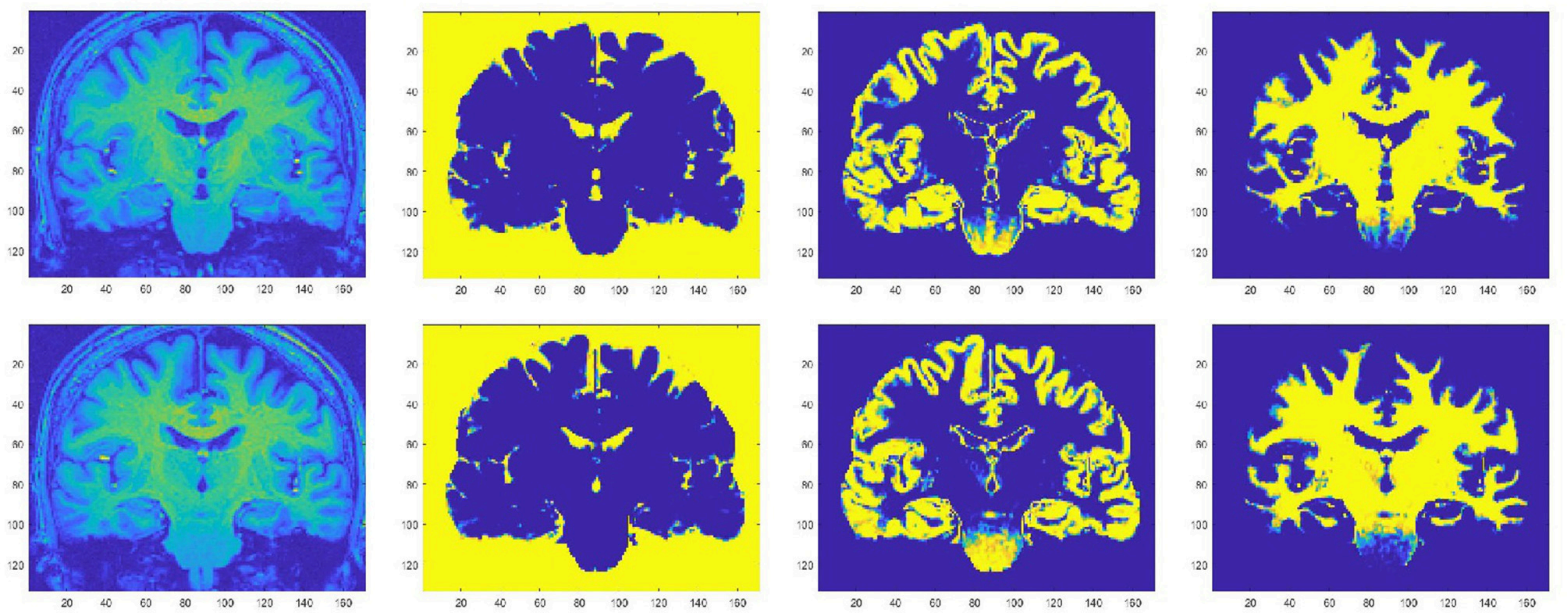
Motor cortical atrophy in a patient with ALS, more pronounced on left side (which correlated with the pattern of weakness clinically). Sequence: 3T, T1w IR, TR 8.4 ms, TE 3.9 ms, TI 1000, FOV 240 mm, Acq voxel 1 × 1 × 1 mm Recon matrix 0.94 × 0.94 × 1 mm. Segmentation algorithm according to Chuang et al. (183).

Focal cortical atrophy has been demonstrated in the precentral gyrus (180–182) (Figure 2), as well as in other motor and non-motor areas, including frontal (181), parietal (184), temporal (185), limbic (186, 187), thalamic (188), bulbar (189), and spinal regions (190). Precentral atrophy predominates in regions of the motor homunculus that correspond to areas most affected by disease (191), whilst frontal cortical atrophy is especially pronounced in patients with associated cognitive dysfunction (192) or fronto-temporal dementia (193). White matter atrophy has been demonstrated in the corticospinal tract (194), front-temporal (192), cerebellar, callosal, and occipital regions (195), but, overall, global atrophy tends to be mild (185).

Atrophy is thought to be a surrogate of neuroaxonal loss (196, 197) and MRI studies have supported the concept that neurodegeneration in ALS is not confined to motor regions. However, volumetric analysis in isolation is not sufficiently sensitive at individual level and, at present, the role of conventional structural MRI in clinical practice is mainly for the exclusion of ALS mimics as part of routine diagnostic workup (198).

Longitudinal studies assessing primary motor cortex (191, 199), subcortical regions (186), and cervical spinal cord (189, 190) have demonstrated worsening atrophy over time, and that the rate of volume loss is greater in rapidly progressive patients, compared to slow progressors (199). Reduction of cervical spinal cord surface area has been shown to correlate with clinical measures of disability, for example ALSFRS-R scores (189), and cervical spinal cord volume decrease over 3 months was predictive of respiratory dysfunction in the subsequent year in one study (190). Cervical atrophy therefore may have potential as a predictive and progression biomarker.

### Signal Changes

High signal may be seen in motor areas on T2-weighted, proton density, or fluid-attenuated inversion recovery (FLAIR) images (199–201), especially in the subcortical precentral white matter and in the posterior limb of the internal capsule. T2 signal change can reflect a number of different underlying mechanisms, for example, oedema, inflammation, demyelination, or, in ALS, most likely neuroaxonal loss or gliosis, either alone or in combination (202, 203), and is neither sensitive nor specific in ALS. T2 signal change in the corticospinal tracts does not appear to correlate well with clinical measures (200, 203).

Cortical hypointensities assessed both qualitatively and quantitatively, on T2-weighted (204), T2^*^-weighted (205), and susceptibility-weighted images (206) are thought to reflect reactive ferritin-laden microglia accumulating in the deep layers of the precentral gyrus (206, 207). Ferritin contains iron which is paramagnetic and alters T2^*^ relaxation, leading to hypointensities on T2-weighted, T2^*^-weighted, and susceptibility-weighted images, a feature that increases with static magnetic field strength. Although these findings were not replicated by another study (208), and such changes appear rather non-specific since they have also been shown in healthy individuals, T2-weighted hypointensities do correlate with UMN signs in ALS patients (207, 209, 210) and can appear early in the disease process (190).

### Diffusion Tensor Imaging (DTI)

Diffusion tensor imaging (DTI) exploits differences in local directionality of water diffusion to assess tissue architecture and is especially suited to the study of white matter tracts. Fractional anisotropy (FA) is a derived measure which can represent tract integrity. In ALS, FA reduction in the corticospinal tracts and corpus callosum is a consistent finding (211–214) which correlates with clinical measures of disease progression (190, 211, 215–217). Associated elevations in mean diffusivity (MD), a scalar measure representing total diffusion within a voxel, have been reported in a number of these studies (211, 218). Low FA has also been demonstrated in the cervical spinal cord (219, 220) and in extra-motor regions (217, 221, 222). Longitudinal reductions in FA over time have been shown in both motor and extra-motor areas (223, 224).

DTI has demonstrated widespread white matter tract damage supporting the concept of ALS as a multi-system disorder. Diagnostic sensitivity and specificity of 68 and 73%, respectively, has been reported (225). Recent work has applied DTI to create *in vivo* disease staging models, to probe hypotheses of pathophysiological spread in ALS (5, 226).

### Combination of Structural MRI and DTI

Machine learning algorithms combining both volumetric gray matter and DTI measures have been reported to discriminate ALS patients from healthy controls with 86% sensitivity, 67% specificity, and 78% accuracy (227), and ALS patients from ALS-mimics with 92% sensitivity, 75% specificity, and 87% accuracy (228).

### Magnetization Transfer Imaging (MTI)

Magnetization can undergo transfer between bound water, macromolecular groups and free MR-observable water. This interaction can be used to provide the tissue contrast exploited in Magnetization Transfer Imaging (MTI), often interpreted as a measure of myelin integrity or neuroaxonal damage. Reduced MTI ratios have been reported in the corticospinal tracts and extra-motor gray matter of patients with ALS compared to controls (229–231) although these findings were not replicated in one report (199).

### Functional Magnetic Resonance Imaging (fMRI)

Blood oxygen level-dependent (BOLD) functional MRI (fMRI) can detect regions of neuronal and synaptic activation in response to experimental stimuli. A localized vascular response to energy use and demand causes “active” regions to receive an increased oxygenated blood supply, and the MR signal is differentially attenuated according to blood oxygenation level. Aspects of brain physiology can therefore be assessed, based on an assumption of neurovascular coupling. Cortical reorganization has been demonstrated in patients with ALS, with increased activation of contralateral and ipsilateral motor areas including sensorimotor cortex, supplementary motor areas, basal ganglia and cerebellum during motor tasks (232–235). Contralateral over-activation correlates with disease progression (236). Reduced activation has been observed in dorsolateral prefrontal cortex (235) and in other studies which investigated tongue movements in patients with bulbar dysfunction (237, 238). Longitudinal studies have demonstrated that increased sensorimotor cortical activation (perhaps attributable to loss of intracortical inhibition) is followed by decreased activation later (probably as motor neurons degenerate) (238). Contrasting results were obtained following motor imagery experiments. Increased activity was seen in patients compared with controls in one study (239), but reduced activity in another (240). In addition to an external stimuli-driven BOLD response, resting state abnormalities have been demonstrated (241). Patients have been shown to demonstrate abnormalities in cerebral regions associated with executive functions (242), and emotional (243, 244), sensory (245), and language (246) processing.

### Magnetic Resonance Spectroscopy

**Figure 3:**
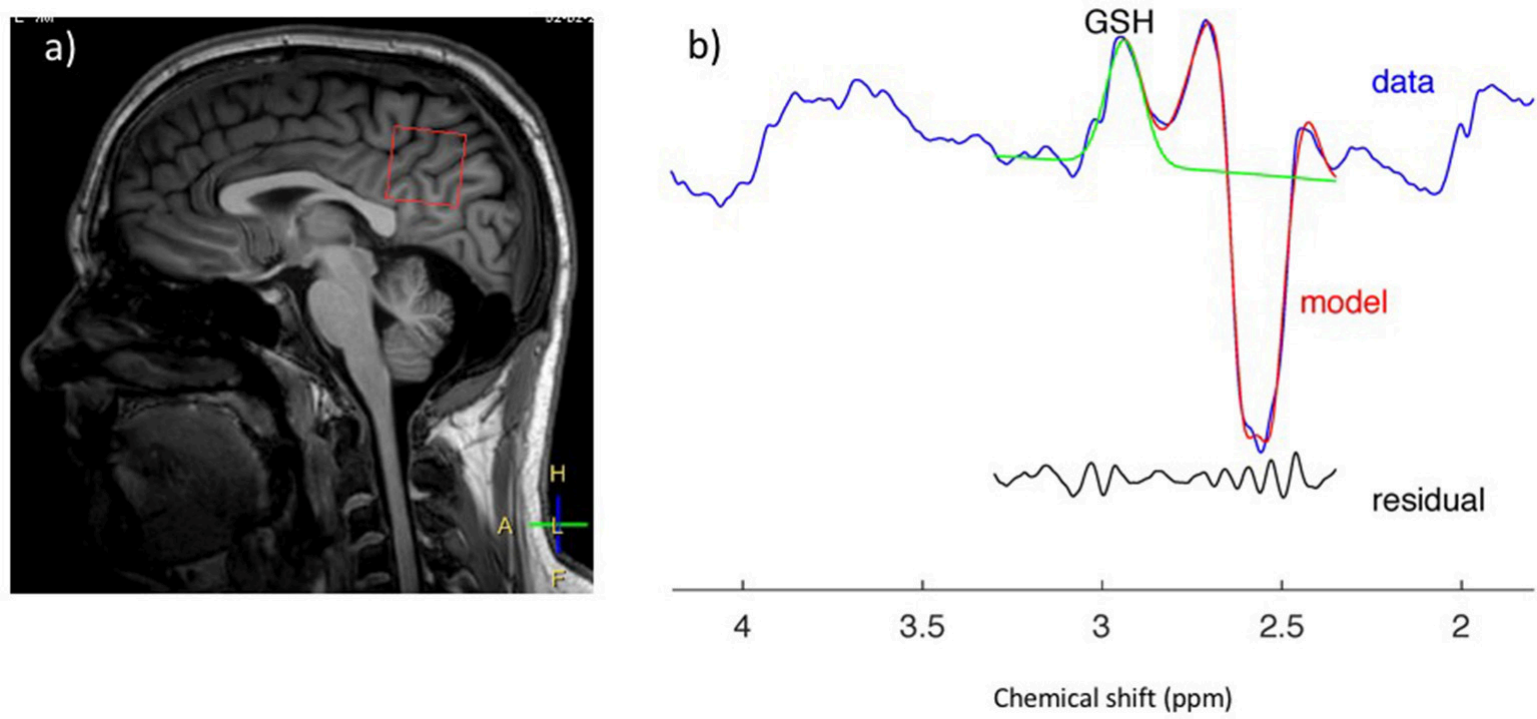
GSH spectrum **(B)** from medial parietal cortex **(A)** (MEGA-PRESS sequence, HERMES spectral editing). **(B)** Green line showing spectral edited GSH peak.

Magnetic resonance spectroscopy (MRS) is a promising advanced MR technique which provides insights into tissue neurobiology through direct measurement of metabolites (Figure 3). Proton spectroscopy of the brain (^1^H-MRS) enables detection of the neuronal molecule N-acetylaspartate (NAA), the glial marker myoinositol (mI), choline-containing compounds (Cho), amino acids and neurotransmitters such as glutamate, glutamine, and gamma-aminobutyric acid (Glu, Gln, and GABA), and creatine, phosphocreatine, and glutathione (Cr, PCr, and GSH) which are compounds related to cellular bioenergetic and oxidative status. Brain ^1^H-MRS studies have demonstrated a widespread reduction in NAA correlating with UMN burden (247–249) in regions spanned by the pyramidal tract (247, 248, 250–265) and in other cortical and subcortical areas (266). NAA has been proposed as an objective indicator of UMN dysfunction and as a potential diagnostic biomarker: sensitivity and specificity of the NAA/Cho ratio have been reported to be 100% and 85% (267), and to be superior to anatomical MRI (268, 269), DTI (267), and transcranial magnetic stimulation (270). The combination of ^1^H-MRS and DTI to diagnose ALS yields a high positive likelihood ratio (6.20) and low negative likelihood ratio (0.08), with potentially useful sensitivity and specificity of 90 and 85%, respectively (271). Although publications assessing longitudinal NAA changes have reached inconsistent conclusions (272–274), NAA concentration has also been used as a marker of treatment response in a number of clinical trials (275–281).

Total creatine (Cr and PCr) appears unchanged (248, 259, 282), but studies measuring Glu, mI, Cho, GABA, and GSH have produced conflicting results and, at present, it is unclear whether the concentration of these molecules is altered in ALS (255, 258, 259, 263, 264, 282).

As highlighted above, published findings from studies that utilize ^1^H-MRS have reported conflicting results at times. In addition to differences between study groups, MR system manufacturer, and spectroscopic analysis methodology, the basic acquisition technique can vary (e.g., echo-generation type, localization method, TR, TE), which may partially explain the lack of consensus. As with standard MRI, the relative contributions from different spectral resonances can be weighted by intrinsic factors such as proton density, T1-, and T2-relaxation rates for each of the metabolites. For ^1^H-MRS, to further our understanding and provide indications of pathophysiology, disease stage and potential therapeutic response, well-characterized and appropriately standardized ^1^H-MRS acquisition methodology is warranted.

## Peripheral Nerve Imaging

In ALS, secondary effects on peripheral nerve are the least studied anatomical location with MRI, but the technique shows potential and has been investigated (283, 284). In a recent longitudinal study, the FA of tibial and peroneal nerve was shown to decrease with disease progression and to correlate with ALSFRS-R, showing potential as a biomarker of disease progression (285).

## Muscle Imaging And Spectroscopy

**Figure 4:**
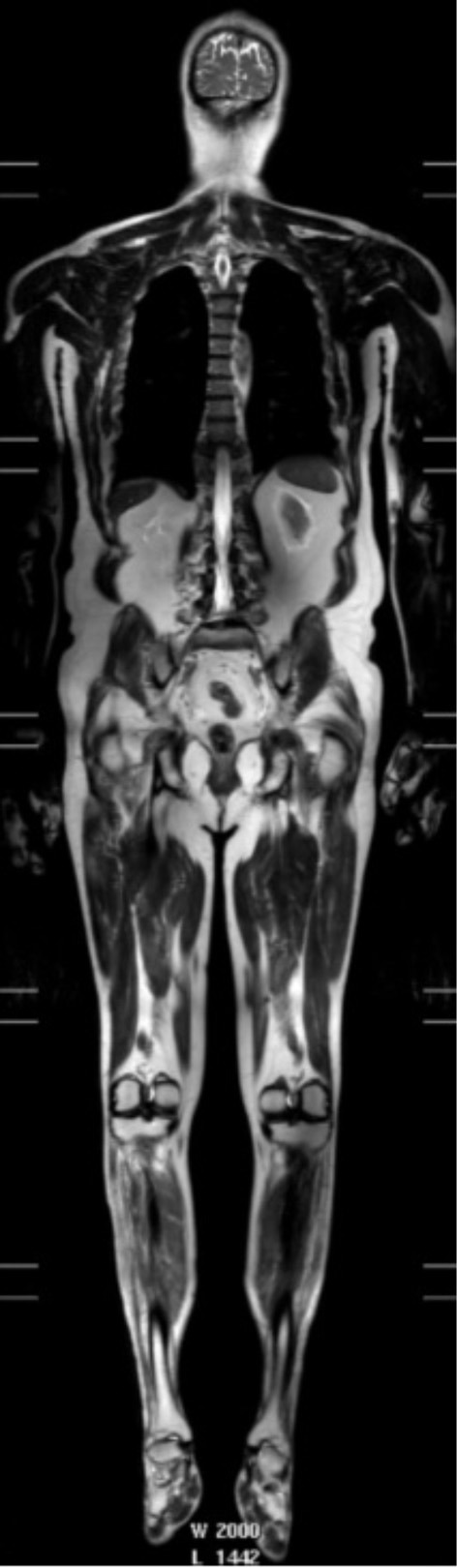
T2-weighted whole body image acquired in a patient: 3T, single shot TSE, TR 1107 ms, TE 80 ms, FOV 37 × 55 cm, voxel size 1.25 × 1.5 × 5 mm recon 0.78 × 0.78 × 5—used with permission from Jenkins et al. (288).

Anterior horn cell denervation in ALS leads to secondary signal change and atrophy in muscles and nerves which can be assessed with MRI and potentially employed as a marker of disease progression. An early study showed reductions in the volume of the tongue in up to two-thirds of ALS patients (284). Tibialis anterior volume reduction and increased T2-relaxation times were observed in a longitudinal study of 11 patients (286) and correlated with clinical (maximal voluntary isometric contraction, MVIC) and electrophysiological (CMAP) measures. Limb muscle signal changes have been demonstrated in cross-sectional studies using qualitative observer assessment scales (283, 287). A more recent longitudinal whole-body muscle MRI assessment demonstrated semi-quantitative T2 changes in multiple body regions in ALS patients compared with controls, as well as associations with clinical power and MUNIX, and longitudinal increases signal changes in the tibialis anterior muscle over 4 months (288) (Figure 4).

Metabolites related to cellular bioenergetics, such as adenosine triphosphate (ATP), PCr, and inorganic phosphate (Pi), as well as intracellular pH, have been measured in muscle using phosphorus-31 spectroscopy (^31^P-MRS); some studies have also employed dynamic protocols to assess PCr and pH variations during muscle contraction. PCr recovery (a parameter that correlates with mitochondrial oxidative capacity) was found to be prolonged in patients in one study (289) but was reported unchanged in another (290). Additionally, there appears to be a decreased drop in PCr upon muscular contraction in ALS patients, likely due to lack of available motor units to recruit (291), although other hypotheses, such as impaired central activation or even existence of ALS related primary muscular changes, have also been proposed (292, 293). The potential of ^31^P-MRS being a putative marker of energy dysmetabolism and disease progression has not yet been fully explored.

## Positron Emission Tomography

Positron emission tomography (PET) is another imaging modality that has been employed primarily to investigate ALS pathophysiology, but has shown some potential as a diagnostic biomarker. Relatively fewer PET studies have been conducted in ALS, possibly because this modality, albeit non-invasive, involves exposure to ionizing radiation, and because radiotracer development is a complex process that requires a cyclotron and a specialized multidisciplinary team.

[18F]Fluoro-2-deoxy-2-D-glucose (FDG) PET measures cellular glucose uptake and can assess metabolic activity of brain regions. In ALS, decreased FDG uptake, a probable corollary of neurodegeneration, has been reported in the motor, premotor, and prefrontal cortices as well as in the basal ganglia (294, 295). Notably, the severity of hypometabolism in the front-temporal cortex was associated with cognitive decline and was predictive of shorter survival (296, 297). Interestingly, increased FDG uptake has also been reported in midbrain, pons, hippocampus, superior temporal gyrus, and cerebellum (295, 298). This could perhaps reflect neuronal hyperexcitability, adaptive cellular changes within metabolically active pathways, and/or astrocytic proliferation (295). These findings further corroborate the hypothesis that ALS-related dysmetabolism does not pertain exclusively to motor areas. In addition, midbrain hypermetabolism appears to be relatively specific to ALS and could potentially be valuable in the diagnostic workup of ALS patients (295, 297). Data on altered glucose uptake in the amygdala, parietal, and occipital cortices is more equivocal: lack of consensus could be due to differences either in study protocols or control groups (299).

Neuroinflammation is considered a potentially important contributor to the pathophysiological cascade in ALS and there have been ongoing efforts to develop immune-modifying therapeutics. In this context, assessment of *in vivo* microglial activation by PET could potentially be employed in clinical trials to provide evidence of target engagement and, possibly, to be used as a biomarker of disease response. Microglial activation can be investigated using radiotracers targeting the 18 kDa translocator protein (TSPO), also known as the peripheral-type benzodiazepine receptor, such as [11C]-(R)-PK11195 (a first generation tracer which is relatively non-specific and has a low signal to background ratio), [18F]-DPA-714, and [11C]-PBR28 (second generation, more specific tracers). TSPO is thought to be expressed specifically by activated microglia and astrocytes. These studies have shown enhanced microglial activation in primary and premotor cortices, prefrontal and temporal cortices, thalamus, and brainstem (300–303). Findings correlated with UMN burden and ALSFRS-R score and were associated with concomitant alterations of the glial marker mI and with DTI and spectroscopic measures of tissue damage (300, 302, 303).

Other work has provided further insights into ALS pathogenesis by showing evidence of inhibitory inter-neuronopathy (employing the GABA-A ligand, [11C]flumazenil) (304, 305), alteration of serotoninergic neurotransmission [using the radiotracer [11C]-WAY100635] (306), and increased oxidative stress [by [62Cu]-ATSM] (307).

In summary, whilst MR and PET studies have made important contributions toward elucidating disease mechanisms *in vivo* in patients with ALS, a fully validated biomarker sensitive and specific to disease change at individual level remains elusive. This represents an important area of need in the field (308).

## Electrophysiology Biomarkers

### Motor Unit Number Estimation (MUNE)

First developed in the 1970s, motor unit number estimation (MUNE) aims to provide a reproducible, quantitative measure of the number of functional motor units (309). Numerous MUNE methods have emerged predominantly based on the same underlying principle. First, a summated value for the total motor unit population within a nerve, the maximum compound muscle action potential (CMAP) amplitude, is obtained. This is then divided by a value representing the average single motor unit in that nerve, thus providing an estimate of motor unit number (309, 310).

MUNE calculations differ in the approach taken to measuring a typical single motor unit (311). For example, the original incremental method utilized the concept of different axons having differing excitation thresholds, with step-wise increases in stimulus intensity used to recruit additional discrete motor units (309). However, subsequent work determined that repeated presentation of the same stimulus may activate different motor axons with similar stimulation thresholds, thus resulting in CMAP changes not representative of single motor unit size, a phenomenon termed alternation (310). The multiple point stimulation (MPS) method (and later adaptations) attempted to circumvent this through stimulation at distinct points along the nerve in an attempt to sample different motor axons (312). Further developments included a multipoint incremental MUNE, combining incremental and MPS methods. This technique had a number of practical advantages over other methods in that it is simple, relatively rapid to perform (~5 min per muscle), well-tolerated (as multiple supramaximal stimuli are not performed), and does not require specialized equipment (313). Statistical approaches to the *post-hoc* analysis of data have also been proposed (314, 315).

Incremental, MPS and multipoint incremental MUNE methods have been reported as reliable and sensitive tools, correlating with and outperforming other functional clinical measures in demonstrating disease progression (249, 316). MPS has additionally been observed to identify preclinical LMN loss (249). Despite promising findings in familial (317) and sporadic cohorts (318), Poisson statistical methods were unable to account for the increased motor unit variability found in patients with ALS (318). Similarly, despite promising initial results (315), dissemination and validation of Bayesian statistical methods, which allow for sources of variability and uncertainty, has been limited by the technically intensive nature of the process (310, 319).

High-density MUNE utilizes a large number of electrode channels to resolve alternation, whilst also enabling the measurement of proximal and distal muscles, a feature not offered by most MUNE techniques (320). The requirement for specific equipment and software has precluded its widespread use thus far (319).

Recently, a novel MUNE method, MScanFIT MUNE (MScan), has been proposed, using detailed stimulus-response curves, or “CMAP scans,” which provide information on all motor units contributing to the CMAP, unlike other MUNE methods (321). Preliminary findings appear promising, demonstrating superior reproducibility, detection of motor unit loss, and disease progression compared to other MUNE methods (322).

**Figure 5:**
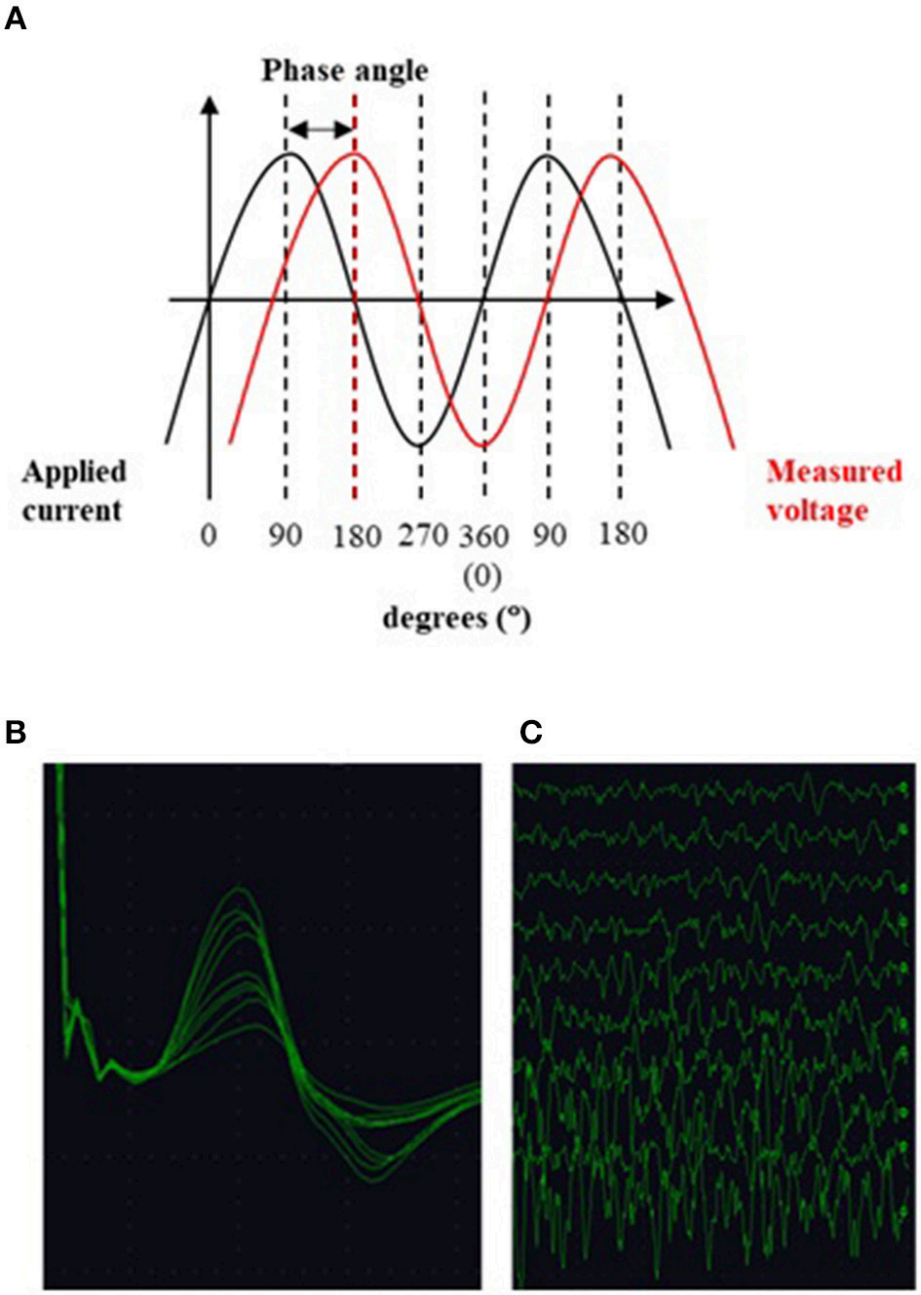
**(A)** EIM: alternating current is applied to the muscle and the ensuing voltage measured. The phase angle is a metric of tissue impedance and describes, in degrees, the angle of asynchrony between the two sinusoidal waveforms. **(B)** Multiple compound muscle action potentials recorded during the incremental motor unit number estimation technique. Each change in amplitude is thought to represent the addition of a new motor unit. **(C)** Surface interference patterns obtained during the motor unit number index technique. After recording a maximal compound muscle action potential the subject performs isometric contraction of the muscle of interest with increasing force. Parameters from these recordings are then used together with data from the CMAP to compute the index.

Motor unit number index (MUNIX) applies a mathematical model based on the CMAP and surface EMG interference pattern at different voluntary activation levels (Figures 5B,C) (323). It overcomes a number of MUNE limitations by enabling fast (< 5 min/muscle), easy to perform measurements of any proximal or distal muscle from which a supramaximal CMAP can be elicited (324).

The sensitivity and reliability of MUNIX as a marker of disease progression in ALS was highlighted in a 15-month longitudinal multicenter study (325), with further work demonstrating a significant correlation between MUNIX and various MUNE techniques (27, 311, 326, 327). The importance of optimizing maximum CMAP amplitude during MUNIX recording has been emphasized (324). MUNIX measurement reliability has been shown to improve when employing a qualification process including face-to-face teaching and training, with ongoing support for evaluators (328).

Despite these promising results, some authors have suggested that values depend too heavily on CMAP amplitude to provide useful estimates of motor unit numbers (326). However, MUNIX has been shown to exhibit superior sensitivity to early change when compared to ALSFRS-R, manual muscle testing, and CMAP amplitude (329). Furthermore, a capacity to detect pre-symptomatic LMN loss has also been reported (330).

Multi-muscle global MUNIX scores have been investigated as a measure of multi-segment involvement (331), allowing more broad evaluation of motor unit loss and insight into the pattern of disease spread (330, 331). Such instruments have shown increased sensitivity to progression when compared to single-muscle MUNIX (331) and ALSFRS-R (325), reducing the time required to detect therapeutic change (325). This approach may, however, over-represent individual segments (331) and negative results have also been reported (332). Nonetheless, MUNIX offers interesting insights into disease progression and is undergoing worldwide evaluation in clinical trials.

### Neurophysiological Index

The neurophysiological index (NI) has been proposed as a quantitative measure of peripheral disease burden in ALS patients. It collectively expresses changes observed during disease progression using standard neurophysiological measures: increases in distal motor latency and F-wave frequency, and a decrease in CMAP amplitude (333, 334). Previous studies report NI to be a reliable measure (335), differentiating fALS and sALS cohorts from healthy controls (336, 337). As a surrogate measure of disease progression, NI has displayed decline at a greater rate (41.9% at 6 months) than ALSFRS-R (18.4%), FVC (15.4%), and CMAP amplitude (25.5%) (316), with sensitivity to change in as little as 4 weeks (329). While further work is needed, the NI has been implemented in clinical trials and has been proposed as a method to expedite completion of future phase II trials (334).

### Axonal Excitability

Axonal excitability measurement techniques allow non-invasive, *in vivo* assessment of the biophysical properties of peripheral axons (338). Employing threshold tracking methods allows sensitivity to changes in the membrane potential caused by activation of ion channels and electrogenic ion pumps (339). Indices used in threshold-tracking axonal excitability testing have provided information of pathological significance in ALS (338). Upregulation of persistent Na^+^ conductances and reduction of slow and fast K^+^ channel conductances have been demonstrated, with the net result being motor axonal hyperexcitability (340).

Axonal ion channel dysfunction has been observed in sALS and fALS cohorts, and supported by mouse models (341–343). Such membrane hyperexcitability is postulated to promote the generation of fasciculations and muscle cramps (341), with intra-axonal Ca^2+^ accumulation due to persistent Na^+^ influx implicated in the neurodegenerative process (338, 344). In keeping with this, changes in axonal excitability have been reported to correlate with more standard measures of motor axon degeneration, such as CMAP amplitude (345). A persistent Na^+^ conductance has been observed to be a predictor for shorter survival time and rapid inter-regional spread (346, 347). Changes in the pattern of abnormal membrane properties with disease progression have also been reported (341). Availability of the specialist hardware/software may limit uptake; however further study of longitudinal utility and test-retest reproducibility is warranted.

### Electrical Impedance Myography (EIM)

Electrical impedance myography (EIM) provides a non-invasive, painless and quantitative method for the evaluation of muscle (Figure 5A). Low-intensity, high-frequency alternating electrical current is applied via surface electrodes to a muscle (or muscle group) of interest and the resulting surface voltages measured. The fundamental basis of EIM is that these recorded surface voltages reflect the conductive and capacitive properties of the underlying tissue, with disease-related changes in muscle morphology, such as muscle fiber atrophy, resulting in altered impedance values (348).

EIM is easy to perform, allows study of proximal and distal muscles, and requires limited subject cooperation and evaluator training (349, 350). It has been shown to be a highly reproducible tool, correlating with established electrophysiologic and functional measures of disease severity (351, 352). Multicenter data have reported sensitivity of EIM to disease progression, demonstrating its potential to expedite phase II clinical trials by reducing the sample size required to detect a treatment effect by more than 50% compared to the ALSFRS-R (353). Evidence for the utility of EIM in the diagnosis of ALS is preliminary, with further study required into its ability to distinguish ALS from other neuromuscular diseases (354).

More recently, EIM has been applied to the evaluation of bulbar dysfunction in ALS, an area of particular importance given the prognostic implications and lack of objective, quantifiable bedside measures of bulbar status (355, 356). Initial investigation has indicated tongue EIM to be a reliable technique, significantly correlating with tongue endurance and the ALSFRS-R bulbar subscore, and distinguishing healthy and diseased muscle (357, 358). Despite the encouraging results emerging principally from a single laboratory, EIM remains in need of development and optimization (355). Further interdisciplinary investigation would allow greater appreciation of the utility of EIM as an objective clinical measure.

### Transcranial Magnetic Stimulation (TMS)

The diagnosis of ALS relies on identification of a combination of UMN and LMN features (359). Conventional electrophysiological techniques objectively assess LMN function. Evaluation of UMN involvement, however, remains solely based on clinical examination (360). Pioneered by Barker and colleagues (361), transcranial magnetic stimulation (TMS) is a non-invasive neurophysiological technique that assesses UMN functional integrity (360, 362). Differences in a number of TMS parameters, signifying a change in cortical excitability, have been identified as an early and specific feature in patients with both sporadic (337) and familial ALS (336). Such abnormalities, including reductions in short-interval intracortical inhibition and cortical silent period duration, and increases in intracortical facilitation and motor evoked potential amplitude (362), precede evidence of LMN dysfunction (363, 364), correlate with measures of peripheral disease burden (337), and relate to the pattern of disease spread (365). These findings provide pathological insight and lend support to the dying-forward hypothesis of ALS as a primary disease of the cortical motor neuron (360).

Recently developed, threshold tracking TMS (TTTMS) (337) has produced important results, including facilitating reliable differentiation of ALS from mimic disorders (366), an improvement in diagnostic sensitivity when compared to the Awaji-Shima criteria, and a reduced time to diagnosis (364). To date, this technique has been largely pioneered by a single group; if reproduced in other centers, the case for incorporation of TTTMS as an objective tool for assessing in future ALS diagnostic criteria would be strong. Evidence supporting the use of TMS as a biomarker assessing longitudinal change is, however, more preliminary and has employed traditional TMS techniques, with conflicting conclusions reached in the ability to monitor disease progression (367, 368), in addition to limited application in ALS therapeutic trials (369). This area remains an exciting field for the ALS community to develop over the coming years.

## Conclusions

The breadth of the research outlined above is an indication of the efforts being undertaken to better understand the pathophysiology of ALS and to discover and validate biomarkers. Common themes occur in each described modality.

Biomarker exploration is dependent on replication. Using biofluid samples as an example, by using agreed SOPs for sample collection and for analysis, more robust conclusions can be drawn. In this way results from multiple centers can be pooled, providing sufficient statistical power to label a biomarker as useful or not. Once a potential biomarker is identified, it can be validated using round-robin or “reverse” round-robin methodology (31). If not successful then a consensus approach should be established to shift focus onto other promising markers. A similar approach in imaging has been established: The Neuroimaging Society in ALS (NiSALS) is a collaboration of neuroimaging scientists to discuss imaging methodologies in the disease as well as providing a solution to the challenge of analyzing MRI data from different sites and protocols (308). Successful validation from meticulous research methodology unfortunately then has the additional hurdle of becoming valid in clinical practice, wherein there is new heterogeneity, with reliance on healthcare professionals and hospital laboratories to collect and process samples in a comparable way.

Despite excellent attempts in each field, single useful biomarkers of ALS are as of yet out of reach. Combining biomarkers within a modality is a useful way to improve their utility, although this increases the risk of false positives, and the more biomarkers that are used the higher the sample number needed to confirm significance (370). Additionally, combining markers across modalities is a logical approach to maximize the strengths and sensitivities of each method. With the vast amount of data that this yields, particularly with the use of “-omic” approaches, machine learning techniques may yield the best combinations to maximize sensitivity and specificity. To this end, collaboration with bioinformaticians is essential.

Collaborative efforts like the Pooled Resource, Open access ALS clinical trials (PRO-ACT) database, provide researchers with a large body of well-categorized, longitudinal, patient data sets. This is especially useful in a relatively rare disease like ALS. It can be used to increase the statistical power during analysis of single biomarkers and for machine learning models. Prize4Life, a non-profit organization, asked for models that best predicted survival based on the PRO-ACT data. Algorithms and machine learning approaches were submitted and shown to improve prediction as compared to clinician assessments, and that these methods could reduce the cost of trials through a reduction in sample size. Additionally, this approach identified features previously unrecognized in their contribution to prediction such as creatine kinase, pulse and blood pressure (371). Other research groups continue to use the PRO-ACT data and have developed models of disease progression (372) and survival (373), and have clarified, for example, the predictive utility of urate as a biomarker (169). Whilst this exercise is undoubtedly useful, the importance of standardized collection and analysis methods remains.

The majority of studies explore diagnostic biomarkers, and many exist contrasting patients with healthy controls. However, if a patient with typical ALS is seen by a neurologist, particularly a neuromuscular specialist, then there is rarely a diagnostic dilemma. Ideally, comparisons should be made between ALS and those patients with disease mimics e.g., multifocal motor neuropathy with conduction block or monomelic amyotrophy. Moreover, as explained above, the survival and disability heterogeneity in ALS is large and to this end longitudinal studies assessing how the disease changes over time, measured through surrogate biomarkers, will provide improved information to better sub-classify patients and their prognosis and ensure trial success.

Future biomarker studies should aim to encapsulate all phenotype data as well as genetic and biological information to help stratification. The above point is well-explained by Benatar et al. (374) and furthermore they outline general points for researchers to be aware of in ALS longitudinal studies. During longitudinal follow-up, studies may enrich with slow-progressors, implying that conclusions that are drawn are not necessarily applicable for the whole population. Secondly, attempting to define disease onset is difficult, given that disease is likely to be active before presentation to healthcare; “baseline” comparisons are therefore not valid. However, most ALS progresses linearly and as such there is value in measuring fixed interval time points from the “recruitment baseline.”

## Author Contributions

PS and NV conceived the concept and structure of the review. NV wrote the sections on introduction, cerebrospinal fluid and conclusion. SS and SM wrote the section on blood and SS wrote the section on urine. HM wrote the section on electrophysiology with oversight from JA. MS wrote the section on imaging with oversight from TJ and IW. All authors reviewed the final manuscript and offered critical feedback.

### Conflict of Interest Statement

The authors declare that the research was conducted in the absence of any commercial or financial relationships that could be construed as a potential conflict of interest.

## References

[1] Woolley SC Jonathan SK. Cognitive and behavioral impairment in amyotrophic lateral sclerosis. Phys Med Rehabil Clin N Am. (2008) 19:607–17. 10.1016/j.pmr.2008.04.002 18625419

[2] Mitchell JD Callagher P Gardham J Mitchell C Dixon M Addison-Jones R. Timelines in the diagnostic evaluation of people with suspected amyotrophic lateral sclerosis (ALS)/motor neuron disease (MND)–a 20-year review: can we do better? Amyotroph Lateral Scler. (2010) 11:537–41. 10.3109/17482968.2010.495158 20565332

[3] Fatima M Tan R Halliday GM Kril JJ. Spread of pathology in amyotrophic lateral sclerosis: assessment of phosphorylated TDP-43 along axonal pathways. Acta Neuropathol Commun. (2015) 3:47. 10.1186/s40478-015-0226-y 26216351 PMC4517552

[4] Brettschneider J Del Tredici K Toledo JB Robinson JL Irwin DJ Grossman M. Stages of pTDP-43 pathology in amyotrophic lateral sclerosis. Ann Neurol. (2013) 74:20–38. 10.1002/ana.23937 23686809 PMC3785076

[5] Kassubek J Muller HP Del Tredici K Brettschneider J Pinkhardt EH Lule D. Diffusion tensor imaging analysis of sequential spreading of disease in amyotrophic lateral sclerosis confirms patterns of TDP-43 pathology. Brain. (2014) 137(Pt. 6):1733–40. 10.1093/brain/awu090 24736303

[6] Kanouchi T Ohkubo T Yokota T. Can regional spreading of amyotrophic lateral sclerosis motor symptoms be explained by prion-like propagation? J Neurol Neurosurg Psychiatry. (2012) 83:739–45. 10.1136/jnnp-2011-301826 22544947 PMC3368493

[7] Ravits J Appel S Baloh RH Barohn R Rix Brooks B Elman L. Deciphering amyotrophic lateral sclerosis: what phenotype, neuropathology and genetics are telling us about pathogenesis. Amyotroph Lateral Scler Frontotemporal Degener. (2013) 14(Suppl. 1):5–18. 10.3109/21678421.2013.778548 23678876 PMC3779649

[8] Westergard T Jensen BK Wen X Cai J Kropf E Iacovitti L. Cell-to-cell transmission of dipeptide repeat proteins linked to C9orf72-ALS/FTD. Cell Rep. (2016) 17:645–52. 10.1016/j.celrep.2016.09.032 27732842 PMC5078984

[9] Spencer PS Palmer VS. Interrelationships of undernutrition and neurotoxicity: food for thought and research attention. Neurotoxicology. (2012) 33:605–16. 10.1016/j.neuro.2012.02.015 22394483 PMC3437940

[10] Desport JC Preux PM Truong TC Vallat JM Sautereau D Couratier P. Nutritional status is a prognostic factor for survival in ALS patients. Neurology. (1999) 53:1059. 10.1212/WNL.53.5.1059 10496266

[11] Marin B Desport JC Kajeu P Jesus P Nicolaud B Nicol M. Alteration of nutritional status at diagnosis is a prognostic factor for survival of amyotrophic lateral sclerosis patients. J Neurol Neurosurg Psychiatry. (2011) 82:628. 10.1136/jnnp.2010.211474 21097551

[12] Bourke SC Tomlinson M Williams TL Bullock RE Shaw PJ Gibson GJ. Effects of non-invasive ventilation on survival and quality of life in patients with amyotrophic lateral sclerosis: a randomised controlled trial. Lancet Neurol. (2006) 5:140–7. 10.1016/S1474-4422(05)70326-4 16426990

[13] Andersen T Sandnes A Vollsæter M Halvorsen T Fondenes O Røksund O Measurement of vital capacity in amyotrophic lateral sclerosis – forced and slowly performed. Eur Resp J. (2015) 46(Suppl. 59):PA3730 10.1183/13993003.congress-2015.PA3730

[14] Czaplinski A Yen AA Appel SH. Forced vital capacity (FVC) as an indicator of survival and disease progression in an ALS clinic population. J Neurol Neurosurg Psychiatry. (2006) 77:390–2. 10.1136/jnnp.2005.072660 16484652 PMC2077717

[15] Polkey MI Lyall RA Yang K Johnson E Leigh PN Moxham J. Respiratory muscle strength as a predictive biomarker for survival in amyotrophic lateral sclerosis. Am J Resp Crit Care Med. (2017) 195:86–95. 10.1164/rccm.201604-0848OC 27494149 PMC5214920

[16] Tilanus TBM Groothuis JT TenBroek-Pastoor JMC Feuth TB Heijdra YF Slenders JPL. The predictive value of respiratory function tests for non-invasive ventilation in amyotrophic lateral sclerosis. Respir Res. (2017) 18:144. 10.1186/s12931-017-0624-8 28743265 PMC5526316

[17] Capozzo R Quaranta VN Pellegrini F Fontana A Copetti M Carratu P Sniff nasal inspiratory pressure as a prognostic factor of tracheostomy or death in amyotrophic lateral sclerosis. J Neurol. (2015) 262:593–603. 10.1007/s00415-014-7613-3 25522696

[18] Stefanutti D Benoist M-R Scheinmann P Chaussain M Fitting J-W Usefulness of sniff nasal pressure in patients with neuromuscular or skeletal disorders. Am J Respir Crit Care Med. (2000) 162:1507–11. 10.1164/ajrccm.162.4.9910034 11029369

[19] Chaudri MB Liu C Watson L Jefferson D Kinnear WJ. Sniff nasal inspiratory pressure as a marker of respiratory function in motor neuron disease. Eur Respir J. (2000) 15:539. 10.1034/j.1399-3003.2000.15.18.x 10759449

[20] Jenkins JAL Sakamuri S Katz JS Forshew DA Guion L Moore D. Phrenic nerve conduction studies as a biomarker of respiratory insufficiency in amyotrophic lateral sclerosis. Amyotroph Lateral Scler Frontotemporal Degener. (2016) 17:213–20. 10.3109/21678421.2015.1112406 26618854

[21] Ahmed RM Newcombe REA Piper AJ Lewis SJ Yee BJ Kiernan MC. (2016). Sleep disorders and respiratory function in amyotrophic lateral sclerosis. Sleep Med Rev. 26:33–42. 10.1016/j.smrv.2015.05.007 26166297

[22] Quaranta VN Carratu P Damiani MF Dragonieri S Capozzolo A Cassano A. The prognostic role of obstructive sleep apnea at the onset of amyotrophic lateral sclerosis. Neurodegen Dis. (2017) 17:14–21. 10.1159/000447560 27595268

[23] Sakka L Coll G Chazal J. Anatomy and physiology of cerebrospinal fluid. Eur Ann Otorhinolaryngol Head Neck Dis. (2011) 128:309–16. 10.1016/j.anorl.2011.03.002 22100360

[24] Rosengren LE Karlsson JE Karlsson JO Persson LI Wikkelso C. Patients with amyotrophic lateral sclerosis and other neurodegenerative diseases have increased levels of neurofilament protein in CSF. J Neurochem. (1996) 67:2013–8. 10.1046/j.1471-4159.1996.67052013.x 8863508

[25] Brettschneider J Petzold A Sussmuth SD Ludolph AC Tumani H. Axonal damage markers in cerebrospinal fluid are increased in ALS. Neurology. (2006) 66:852–6. 10.1212/01.wnl.0000203120.85850.54 16567701

[26] Li D Shen D Tai H Cui L. Neurofilaments in CSF as diagnostic biomarkers in motor neuron disease: a meta-analysis. Front Aging Neurosci. 8:290. 10.3389/fnagi.2016.00290 27965574 PMC5126108

[27] Steinacker P Feneberg E Weishaupt J Brettschneider J Tumani H Andersen PM Neurofilaments in the diagnosis of Moto neuron diseases: a prospective study on 455 patients. J Neurol Neurosurg Psychiatry. (2016) 87:12–20. 10.1136/jnnp-2015-311387 26296871

[28] Posen K De Schaepdryver M Stubendorff B Gille B Muckova P Wendler S Neurofilament markers for ALS correlate with extent of upper and lower motor neuron disease. Neurology. (2017) 88:2302–9. 10.1212/WNL.0000000000004029 28500227

[29] Oeckl P Jardel C Salachas F Lamari F Andersen PM Bowser R. Multicenter validation of CSF neurofilaments as diagnostic biomarkers for ALS. Amyotroph Lateral Scler Frontotemporal Degener. (2016) 17:404–13. 10.3109/21678421.2016.1167913 27415180

[30] Ganesalingam J An J Bowser R Andersen PM Shaw CE. pNfH is a promising biomarker for ALS. Amyotroph Lateral Scler Frontotemporal Degener. (2013) 14:146–9. 10.3109/21678421.2012.729596 23134506

[31] Lehnert S Costa J de Carvalho M Kirby J Kuzma-Kozakiewicz M Morelli C. Multicentre quality control evaluation of different biomarker candidates for amyotrophic lateral sclerosis. Amyotrop Lateral Scler Frontotemporal Degener. (2014) 15:344–50. 10.3109/21678421.2014.884592 24575871

[32] Gaiani A Martinelli I Bello L Querin G Puthenparampil M Ruggero S. Diagnostic and prognostic biomarkers in amyotrophic lateral sclerosis: neurofilament light chain levels in definite subtypes of disease. JAMA Neurol. (2017) 74:525–32. 10.1001/jamaneurol.2016.5398 28264096 PMC5822207

[33] Skillbäck T Mattsson N Blennow K Zetterberg H. Cerebrospinal fluid neurofilament light concentration in motor neuron disease and frontotemporal dementia predicts survival. Amyotroph Lateral Scler Frontotemporal Degener. (2017) 18:397–403. 10.1080/21678421.2017.1281962 28631955

[34] Lu C-H Macdonald-Wallis C Gray E Pearce N Petzold A Norgren N. Neurofilament light chain: a prognostic biomarker in amyotrophic lateral sclerosis. Neurology. (2015) 84:2247–57. 10.1212/WNL.0000000000001642 25934855 PMC4456658

[35] Tortelli R Ruggieri M Cortese R D'Errico E Capozzo R Leo A. Elevated cerebrospinal fluid neurofilament light levels in patients with amyotrophic lateral sclerosis: a possible marker of disease severity and progression. Eur J Neurol. (2012) 19:1561–7. 10.1111/j.1468-1331.2012.03777.x 22680408

[36] Xu Z Henderson RD David M McCombe PA. Neurofilaments as biomarkers for amyotrophic lateral sclerosis: a systematic review and meta-analysis. PLoS ONE. (2016) 11:e0164625. 10.1371/journal.pone.0164625 27732645 PMC5061412

[37] Bourbouli M Rentzos M Bougea A Zouvelou V Constantinides VC Zaganas I. Cerebrospinal fluid TAR DNA-binding protein 43 combined with tau proteins as a candidate biomarker for amyotrophic lateral sclerosis and frontotemporal dementia spectrum disorders. Dementia Geriatr Cogn Disord. (2017) 44:144–52. 10.1159/000478979 28848086

[38] Wilke C Deuschle C Rattay TW Maetzler W Synofzik M Total tau is increased, but phosphorylated tau not decreased, in cerebrospinal fluid in amyotrophic lateral sclerosis. Neurobiol Aging. (2015) 36:1072–4. 10.1016/j.neurobiolaging.2014.10.019 25453560

[39] Paladino P Valentino F Piccoli T Piccoli F La Bella V Cerebrospinal fluid tau protein is not a biological marker in amyotrophic lateral sclerosis. Eur J Neurol. (2009) 16:257–61. 10.1111/j.1468-1331.2008.02405.x 19138331

[40] Neumann M Sampathu DM Kwong LK Truax AC Micsenyi MC Chou TT. Ubiquitinated TDP-43 in frontotemporal lobar degeneration and amyotrophic lateral sclerosis. Science. (2006) 314:130–3. 10.1126/science.1134108 17023659

[41] Mackenzie IR Bigio EH Ince PG Geser F Neumann M Cairns NJ. Pathological TDP-43 distinguishes sporadic amyotrophic lateral sclerosis from amyotrophic lateral sclerosis with SOD1 mutations. Ann Neurol. (2007) 61:427–34. 10.1002/ana.21147 17469116

[42] Steinacker P Hendrich C Sperfeld AD Jesse S von Arnim CAF Lehnert S. TDP-43 in cerebrospinal fluid of patients with frontotemporal lobar degeneration and amyotrophic lateral sclerosis. Arch Neurol. (2008) 65:1481–7. 10.1001/archneur.65.11.1481 19001167 PMC2690860

[43] Kasai T Tokuda T Ishigami N Sasayama H Foulds P Mitchell DJ. Increased TDP-43 protein in cerebrospinal fluid of patients with amyotrophic lateral sclerosis. Acta Neuropathol. (2009) 117:55–62. 10.1007/s00401-008-0456-1 18989684

[44] Noto Y Shibuya K Sato Y Kanai K Misawa S Sawai S. Elevated CSF TDP-43 levels in amyotrophic lateral sclerosis: specificity, sensitivity, and a possible prognostic value. Amyotroph Lateral Scler. (2011) 12:140–3. 10.3109/17482968.2010.541263 21126161

[45] Junttila A Kuvaja M Hartikainen P Siloaho M Helisalmi S Moilanen V. Cerebrospinal fluid TDP-43 in frontotemporal lobar degeneration and amyotrophic lateral sclerosis patients with and without the C9ORF72 hexanucleotide expansion. Dement Geriatr Cogn Disord Extra. (2016) 6:142–9. 10.1159/000444788 27195002 PMC4868946

[46] Feneberg E Steinacker P Lehnert S Schneider A Walther P Thal DR. Limited role of free TDP-43 as a diagnostic tool in neurodegenerative diseases. Amyotroph Lateral Scler Frontotemporal Degener. (2014) 15:351–6. 10.3109/21678421.2014.905606 24834468

[47] Krüger T Lautenschläger J Grosskreutz J Rhode H. Proteome analysis of body fluids for amyotrophic lateral sclerosis biomarker discovery. Proteomics Clin Appl. (2013) 7:123–35. 10.1002/prca.201200067 23129563

[48] Barschke P Oeckl P Steinacker P Ludolph A Otto M. Proteomic studies in the discovery of cerebrospinal fluid biomarkers for amyotrophic lateral sclerosis. Exp Rev Proteomics. (2017) 14:769–77. 10.1080/14789450.2017.1365602 28799854

[49] Collins MA An J Hood BL Conrads TP Bowser RP. Label-Free LC-MS/MS proteomic analysis of cerebrospinal fluid identifies protein/pathway alterations and candidate biomarkers for amyotrophic lateral sclerosis. J Proteome Res. (2015) 14:4486–501. 10.1021/acs.jproteome.5b00804 26401960 PMC5592736

[50] Chen X Chen Y Wei Q Ou R Cao B Zhao B. Assessment of a multiple biomarker panel for diagnosis of amyotrophic lateral sclerosis. BMC Neurol. (2016) 16:173. 10.1186/s12883-016-0689-x 27634542 PMC5024522

[51] Wilson ME Boumaza I Lacomis D Bowser R. Cystatin C: a candidate biomarker for amyotrophic lateral sclerosis. PLoS ONE. (2010) 5:e15133. 10.1371/journal.pone.0015133 21151566 PMC3000338

[52] Ryberg H An J Darko S Lustgarten JL Jaffa M Gopalakrishnan V. Discovery and verification of amyotrophic lateral sclerosis biomarkers by proteomics. Muscle Nerve. (2010) 42:104–11. 10.1002/mus.21683 20583124 PMC2975276

[53] Ren Y Zhu W Cui F Yang F Chen Z Ling L. Measurement of cystatin C levels in the cerebrospinal fluid of patients with amyotrophic lateral sclerosis. Int J Clin Exp Pathol. (2015) 8:5419–26. Available online at: http://www.ijcep.com/26191245 PMC4503116

[54] Varghese AM Sharma A Mishra P Vijayalakshmi K Harsha HC Sathyaprabha TN. Chitotriosidase - a putative biomarker for sporadic amyotrophic lateral sclerosis. Clin Proteomics. (2013) 10:19. 10.1186/1559-0275-10-19 24295388 PMC4220794

[55] Chen Y Liu X-H Wu J-J Ren H-M Wang J Ding Z-T. Proteomic analysis of cerebrospinal fluid in amyotrophic lateral sclerosis. Exp Ther Med. (2016) 11:2095–106. 10.3892/etm.2016.3210 27284291 PMC4887813

[56] Steinacker P Verde F Fang L Feneberg E Oeckl P Roeber S. Chitotriosidase (CHIT1) is increased in microglia and macrophages in spinal cord of amyotrophic lateral sclerosis and cerebrospinal fluid levels correlate with disease severity and progression. J Neurol Neurosurg Psychiatry. (2018) 89:239–47. 10.1136/jnnp-2017-317138 29142138

[57] Thompson AG Gray E Thezenas ML Charles PD Evetts S Hu MT. Cerebrospinal fluid macrophage biomarkers in amyotrophic lateral sclerosis. Ann Neurol. (2018) 83:258–68. 10.1002/ana.25143 29331073

[58] Blasco H Corcia P Moreau C Veau S Fournier C Vourc'h P H-NMR-based metabolomic profiling of CSF in early amyotrophic lateral sclerosis. PLoS ONE. (2010) 5:e13223 10.1371/annotation/2c2f8fce-a5be-40a3-af8f-48f119b2c593 20949041 PMC2951909

[59] Blasco H Nadal-Desbarats L Pradat P-F Gordon PH Antar C Veyrat-Durebex C. Untargeted 1H-NMR metabolomics in CSF. Neurology. (2014) 82:1167–74. 10.1212/WNL.0000000000000274 24587475

[60] Blasco H Veyrat-Durebex C Bocca C Patin F Vourc'h P Kouassi Nzoughet J. Lipidomics reveals cerebrospinal-fluid signatures of ALS. Sci Rep. (2017) 7:17652. 10.1038/s41598-017-17389-9 29247199 PMC5732162

[61] Blasco H Patin F Madji Hounoum B Gordon PH Vourc'h P Andres CR. Metabolomics in amyotrophic lateral sclerosis: how far can it take us? Eur J Neurol. (2016) 23:447–54. 10.1111/ene.12956 26822316

[62] Kumar A Bala L Kalita J Misra UK Singh RL Khetrapal CL. Metabolomic analysis of serum by, H NMR spectroscopy in amyotrophic lateral sclerosis. Clin Chim Acta. (2010) 411:563–7. 10.1016/j.cca.2010.01.016 20096678

[63] Rozen S Cudkowicz ME Bogdanov M Matson WR Kristal BS Beecher C. Metabolomic analysis and signatures in motor neuron disease. Metabolomics. (2005) 1:101–8. 10.1007/s11306-005-4810-1 18820733 PMC2553219

[64] D'Amico E Factor-Litvak P Santella RM Mitsumoto H. Clinical perspective of oxidative stress in sporadic amyotrophic lateral sclerosis. Free Radic Biol Med. (2013) 65:509–27. 10.1016/j.freeradbiomed.2013.06.029 23797033 PMC3859834

[65] Simpson EP Yen AA Appel SH. Oxidative stress: a common denominator in the pathogenesis of amyotrophic lateral sclerosis. Curr Opin Rheumatol. (2003) 15:730–6. 10.1097/00002281-200311000-00008 14569202

[66] Barber SC Shaw PJ. Oxidative stress in ALS: key role in motor neuron injury and therapeutic target. Free Radic Biol Med. (2010) 48:629–41. 10.1016/j.freeradbiomed.2009.11.018 19969067

[67] Rothstein JD. Current hypotheses for the underlying biology of amyotrophic lateral sclerosis. Ann Neurol. (2009) 65:S3–9. 10.1002/ana.21543 19191304

[68] Zetterstrom P Andersen PM Brannstrom T Marklund SL. Misfolded superoxide dismutase-1 in CSF from amyotrophic lateral sclerosis patients. J Neurochem. (2011) 117:91–9. 10.1111/j.1471-4159.2011.07177.x 21226712

[69] Winer L Srinivasan D Chun S Lacomis D Jaffa M Fagan A. SOD1 in cerebral spinal fluid as a pharmacodynamic marker for antisense oligonucleotide therapy. JAMA Neurol. (2013) 70:201–7. 10.1001/jamaneurol.2013.593 23147550 PMC3812918

[70] Miller T Pestronk A David W Rothstein J Simpson E Appel SH Cudkowicz ME A Phase I, randomised, first-in-human study of an antisense oligonucleotide directed against SOD1 delivered intrathecally in SOD1-familial ALS patients. Lancet Neurol. (2013) 12:435–42. 10.1016/S1474-4422(13)70061-9 23541756 PMC3712285

[71] Bali T Self W Liu J Siddique T Wang LH Bird TD. Defining SOD1 ALS natural history to guide therapeutic clinical trial design. J Neurol Neurosurg Psychiatry. (2017) 88:99–105. 10.1136/jnnp-2016-313521 27261500 PMC5136332

[72] Mitsumoto H Santella RM Liu X Bogdanov M Zipprich J Wu HC. Oxidative stress biomarkers in sporadic ALS. Amyotroph Lateral Scler. (2008) 9:177–83. 10.1080/17482960801933942 18574762 PMC4332387

[73] Beal MF Ferrante RJ Browne SE Matthews RT Kowall NW Brown R.H Jr. Increased 3-nitrotyrosine in both sporadic and familial amyotrophic lateral sclerosis. Ann Neurol. (1997) 42:644–54. 10.1002/ana.410420416 9382477

[74] Bogdanov M Brown RH Matson W Smart R Hayden D O'Donnell H. Increased oxidative damage to DNA in ALS patients. Free Radic Biol Med. (2000) 29:652–8. 11033417 10.1016/s0891-5849(00)00349-x

[75] Simpson EP Henry YK Henkel JS Smith RG Appel SH. Increased lipid peroxidation in sera of ALS patients. Neurology. (2004) 62:1758–65. 10.1212/WNL.62.10.1758 15159474

[76] Johnson DA Johnson JA Nrf2 -a therapeutic target for the treatment of neurodegenerative diseases. Free Radic Biol Med. (2015) 88(Pt. B):253–67. 10.1016/j.freeradbiomed.2015.07.147 PMC4809057 26281945

[77] Mead RJ Higginbottom A Allen SP Kirby J Bennett E Barber SC. S[+] Apomorphine is a CNS penetrating activator of the Nrf2-ARE pathway with activity in mouse and patient fibroblast models of amyotrophic lateral sclerosis. Free Radic Biol Med. (2013) 61:438–52. 10.1016/j.freeradbiomed.2013.04.018 23608463 PMC3684770

[78] Ehrhart J Smith AJ Kuzmin-Nichols N Zesiewicz TA Jahan I Shytle RD. Humoral factors in ALS patients during disease progression. J Neuroinflamm. (2015) 12:127. 10.1186/s12974-015-0350-4 26126965 PMC4487852

[79] Siciliano G Piazza S Carlesi C Del Corona A Franzini M Pompella A. Antioxidant capacity and protein oxidation in cerebrospinal fluid of amyotrophic lateral sclerosis. J Neurol. (2007) 254:575–80. 10.1007/s00415-006-0301-1 17426914

[80] Opacic M Stevic Z Bascarevic V Zivic M Spasic M Spasojevic I. Can oxidation-reduction potential of cerebrospinal fluid be a monitoring biomarker in amyotrophic lateral sclerosis? Antioxid Redox Signal. (2017) 28:1570–5. 10.1089/ars.2017.7433 29113448

[81] Malaspina A Puentes F Amor S. Disease origin and progression in amyotrophic lateral sclerosis: an immunology perspective. Int Immunol. (2015) 27:117–29. 10.1093/intimm/dxu099 25344935

[82] Mitchell RM Freeman WM Randazzo WT Stephens HE Beard JL Simmons Z. A CSF biomarker panel for identification of patients with amyotrophic lateral sclerosis. Neurology. (2009) 72:14–9. 10.1212/01.wnl.0000333251.36681.a5 18987350

[83] Guo J Yang X Gao L Zang D. Evaluating the levels of CSF and serum factors in ALS. Brain Behav. (2017) 7:e00637. 10.1002/brb3.637 28293476 PMC5346523

[84] Lind A-L Wu D Freyhult E Bodolea C Ekegren T Larsson A. A multiplex protein panel applied to cerebrospinal fluid reveals three new biomarker candidates in ALS but none in neuropathic pain patients. PLoS ONE. (2016) 11:e0149821. 10.1371/journal.pone.0149821 26914813 PMC4767403

[85] Su XW Clardy SL Stephens HE Simmons Z Connor JR. Serum ferritin is elevated in amyotrophic lateral sclerosis patients. Amyotroph Lateral Scler Frontotemporal Degener. (2015) 16:102–7. 10.3109/21678421.2014.984723 25521651

[86] Liu J Gao L Zang D. Elevated levels of IFN-γ in CSF and serum of patients with amyotrophic lateral sclerosis. PLoS ONE. (2015) 10:e0136937. 10.1145/2818302 26332465 PMC4557946

[87] Cooper-Knock J Walsh MJ Higginbottom A Robin Highley J Dickman MJ Edbauer D. Sequestration of multiple RNA recognition motif-containing proteins by C9orf72 repeat expansions. Brain. (2014) 137(Pt. 7):2040–51. 10.1093/brain/awu120 24866055 PMC4065024

[88] Su Z Zhang Y Gendron TF Bauer PO Chew J Yang W-Y. Discovery of a biomarker and lead small molecules to target r(GGGGCC)-associated defects in c9FTD/ALS. Neuron. (2014) 83:1043–50. 10.1016/j.neuron.2014.07.041 25132468 PMC4232217

[89] Lehmer C Oeckl P Weishaupt JH Volk AE Diehl-Schmid J Schroeter ML. Poly-GP in cerebrospinal fluid links C9orf72-associated dipeptide repeat expression to the asymptomatic phase of ALS/FTD. EMBO Mol Med. (2017) 9:859–68. 10.15252/emmm.201607486 28408402 PMC5494528

[90] Gendron TF Chew J Stankowski JN Hayes LR Zhang Y-J Prudencio M. Poly(GP) proteins are a useful pharmacodynamic marker for C9ORF72-associated amyotrophic lateral sclerosis. Sci Transl Med. 9:eaai7866. 10.1126/scitranslmed.aai7866 28356511 PMC5576451

[91] Waller R Wyles M Heath PR Kazoka M Wollff H Shaw PJ. Small RNA sequencing of sporadic amyotrophic lateral sclerosis cerebrospinal fluid reveals differentially expressed miRNAs related to neural and glial activity. Front Neurosci. 11:731. 10.3389/fnins.2017.00731 29375285 PMC5767269

[92] Freischmidt A Müller K Ludolph AC Weishaupt JH. Systemic dysregulation of TDP-43 binding microRNAs in amyotrophic lateral sclerosis. Acta Neuropathol Commun. (2013) 1:42. 10.1186/2051-5960-1-42 24252274 PMC3893596

[93] De Felice B Annunziata A Fiorentino G Borra M Biffali E Coppola C miR-338-3p is over-expressed in blood, CFS, serum and spinal cord from sporadic amyotrophic lateral sclerosis patients. Neurogenetics. (2014) 15:243–53. 10.1007/s10048-014-0420-2 25130371

[94] Benigni M Ricci C Jones AR Giannini F Al-Chalabi A Battistini S. Identification of miRNAs as Potential biomarkers in cerebrospinal fluid from amyotrophic lateral sclerosis patients. Neuromol Med. (2016) 18:551–60. 10.1007/s12017-016-8396-8 27119371

[95] Juneja T Pericak-Vance MA Laing NG Dave S Siddique T. Prognosis in familial amyotrophic lateral sclerosis: progression and survival in patients with glu100gly and ala4val mutations in Cu,Zn superoxide dismutase. Neurology. (1997) 48:55–7. 10.1212/WNL.48.1.55 9008494

[96] Gaastra B Shatunov A Pulit S Jones AR Sproviero W Gillett A. Rare genetic variation in UNC13A may modify survival in amyotrophic lateral sclerosis. Amyotroph Lateral Scler Frontotemporal Degener. (2016) 17:593–9. 10.1080/21678421.2016.1213852 27584932 PMC5125285

[97] Al-Chalabi A van den Berg LH Veldink J. Gene discovery in amyotrophic lateral sclerosis: implications for clinical management. Nat Rev Neurol. (2017) 13:96–104. 10.1038/nrneurol.2016.182 27982040

[98] Lange DJ Shahbazi M Silani V Ludolph AC Weishaupt JH Ajroud-Driss S. Pyrimethamine significantly lowers cerebrospinal fluid Cu/Zn superoxide dismutase in amyotrophic lateral sclerosis patients with SOD1 mutations. Ann Neurol. (2017) 81:837–48. 10.1002/ana.24950 28480639 PMC5518287

[99] Lange DJ Andersen PM Remanan R Marklund S Benjamin D. Pyrimethamine decreases levels of SOD1 in leukocytes and cerebrospinal fluid of ALS patients: a phase I pilot study. Amyotroph Lateral Scler Frontotemporal Degener. (2013) 14:199–204. 10.3109/17482968.2012.724074 22985433

[100] Cereda C Leoni E Milani P Pansarasa O Mazzini G Guareschi S. Altered intracellular localization of SOD1 in leukocytes from patients with sporadic amyotrophic lateral sclerosis. PLoS ONE. (2013) 8:e75916. 10.1371/journal.pone.0075916 24155874 PMC3796534

[101] De Marco G Lupino E Calvo A Moglia C Buccinna B Grifoni S Cytoplasmic accumulation of TDP-43 in circulating lymphomonocytes of ALS patients with and without TARDBP mutations. Acta Neuropathol. (2011) 121:611–22. 10.1007/s00401-010-0786-7 21120508

[102] Nardo G Pozzi S Pignataro M Lauranzano E Spano G Garbelli S. Amyotrophic lateral sclerosis multiprotein biomarkers in peripheral blood mononuclear cells. PLoS ONE. (2011) 6:e25545. 10.1371/journal.pone.0025545 21998667 PMC3187793

[103] Verstraete E Kuiperij HB van Blitterswijk MM Veldink JH Schelhaas HJ van den Berg LH. TDP-43 plasma levels are higher in amyotrophic lateral sclerosis. Amyotroph Lateral Scler. (2012) 13:446–51. 10.3109/17482968.2012.703208 22873561

[104] Houseman EA Kim S Kelsey KT Wiencke JK. DNA methylation in whole blood: uses and challenges. Curr Environ Health Rep. (2015) 2:145–54. 10.1007/s40572-015-0050-3 26231364

[105] Tremolizzo L Messina P Conti E Sala G Cecchi M Airoldi L. Whole-blood global DNA methylation is increased in amyotrophic lateral sclerosis independently of age of onset. Amyotroph Lateral Scler Frontotemporal Degener. (2014) 15:98–105. 10.3109/21678421.2013.851247 24224837

[106] Coppedè F Stoccoro A Mosca L Gallo R Tarlarini C Lunetta C Increase in DNA methylation in patients with amyotrophic lateral sclerosis carriers of not fully penetrant SOD1 mutations. Amyotroph Lateral Scler Frontotemporal Degener. (2018) 19:93–100. 10.1080/21678421.2017.1367401 28859526

[107] Garton FC Benyamin B Zhao Q Liu Z Gratten J Henders AK. Whole exome sequencing and DNA methylation analysis in a clinical amyotrophic lateral sclerosis cohort. Mol Genet Genomic Med. (2017) 5:418–28. 10.1002/mgg3.302 28717666 PMC5511806

[108] Fogh I Lin K Tiloca C Rooney J Gellera C Diekstra FP. Association of a locus in the CAMTA1 gene with survival in patients with sporadic amyotrophic lateral sclerosis. JAMA Neurol. (2016) 73:812–20. 10.1001/jamaneurol.2016.1114 27244217 PMC5556366

[109] Xi Z Zhang M Bruni AC Maletta RG Colao R Fratta P. The C9orf72 repeat expansion itself is methylated in ALS and FTLD patients. Acta Neuropathol. (2015) 129:715–27. 10.1007/s00401-015-1401-8 25716178

[110] Liu EY Russ J Wu K Neal D Suh E McNally AG. C9orf72 hypermethylation protects against repeat expansion-associated pathology in ALS/FTD. Acta Neuropathol. (2014) 128:525–41. 10.1007/s00401-014-1286-y 24806409 PMC4161616

[111] Gijselinck I Van Mossevelde S Van Der Zee J Sieben A Engelborghs S De Bleecker J. The C9orf72 repeat size correlates with onset age of disease, DNA methylation and transcriptional downregulation of the promoter. Mol Psychiatry. (2016) 21:1112–24. 10.1038/mp.2015.159 26481318 PMC4960451

[112] Liu C Jiang R Yi X Zhu W Bu B. Role of diffusion tensor imaging or magnetic resonance spectroscopy in the diagnosis and disability assessment of amyotrophic lateral sclerosis. J Neurol Sci. (2015) 348:206–10. 10.1016/j.jns.2014.12.004 25524526

[113] Young PE Jew SK Buckland ME Pamphlett R Suter CM. Epigenetic differences between monozygotic twins discordant for amyotrophic lateral sclerosis (ALS) provide clues to disease pathogenesis. PLoS ONE. (2017) 12:1–19. 10.1371/journal.pone.0182638 28797086 PMC5552194

[114] Feneberg E Oeckl P Steinacker P Verde F Barro C Van Damme P. Multicenter evaluation of neurofilaments in early symptom onset amyotrophic lateral sclerosis. Neurology. (2018) 90:e22–30. 10.1212/WNL.0000000000004761 29212830

[115] Ganesalingam J An J Shaw CE Shaw G Lacomis D Bowser R. Combination of neurofilament heavy chain and complement C3 as CSF biomarkers for ALS. J Neurochem. (2011) 117:528–37. 10.1111/j.1471-4159.2011.07224.x 21418221 PMC3076545

[116] De Schaepdryver M Jeromin A Gille B Claeys KG Herbst V Brix B Comparison of elevated phosphorylated neurofilament heavy chains in serum and cerebrospinal fluid of patients with amyotrophic lateral sclerosis. J Neurol Neurosurg Psychiatry. (2017) 89:367–73. 10.1136/jnnp-2017-316605 29054919

[117] Gendron T Bieniek K Zhang Y-J Jansen-West K Ash P Caulfield T. Antisense transcripts of the expanded C9ORF72 hexanucleotide repeat form nuclear RNA foci and undergo repeat-associated non-ATG translation in c9FTD/ALS. Acta Neuropathol. (2013) 126:829–44. 10.1007/s00401-013-1192-8 24129584 PMC3830741

[118] McCombe PA Pfluger C Singh P Lim CYH Airey C Henderson RD. Serial measurements of phosphorylated neurofilament-heavy in the serum of subjects with amyotrophic lateral sclerosis. J Neurol Sci. (2015) 353:122–9. 10.1016/j.jns.2015.04.032 25958264

[119] Gustafson MP Staff NP Bornschlegl S Butler GW Maas L Kazamel M. Comprehensive immune profiling reveals substantial immune system alterations in a subset of patients with amyotrophic lateral sclerosis. PLoS ONE. (2017) 12:e0182002. 10.1371/journal.pone.0182002 28742871 PMC5526569

[120] Murdock BJ Zhou T Kashlan SR Little RJ Goutman SA Feldman EL. Correlation of peripheral immunity with rapid amyotrophic lateral sclerosis progression. JAMA Neurol. (2017) 74:1446–54. 10.1001/jamaneurol.2017.2255 28973548 PMC5822195

[121] Henkel JS Beers DR Wen S Rivera AL Toennis KM Appel JE. Regulatory T-lymphocytes mediate amyotrophic lateral sclerosis progression and survival. EMBO Mol Med. (2013) 5:64–79. 10.1002/emmm.201201544 23143995 PMC3569654

[122] Beers DR Henkel JS Zhao W Wang J Huang A Wen S. Endogenous regulatory T lymphocytes ameliorate amyotrophic lateral sclerosis in mice and correlate with disease progression in patients with amyotrophic lateral sclerosis. Brain. (2011) 134:1293–314. 10.1093/brain/awr074 21596768 PMC3097891

[123] Lu CH Allen K Oei F Leoni E Kuhle J Tree T. Systemic inflammatory response and neuromuscular involvement in amyotrophic lateral sclerosis. Neurol Neuroimmunol Neuroinflamm. (2016) 3:e244. 10.1212/NXI.0000000000000244 27308305 PMC4897985

[124] Poloni M Facchetti D Mai R Micheli A Agnoletti L Francolini G. Circulating levels of tumour necrosis factor-alpha and its soluble receptors are increased in the blood of patients with amyotrophic lateral sclerosis. Neurosci Lett. (2000) 287:211–4. 10.1016/S0304-3940(00)01177-0 10863032

[125] Cereda C Baiocchi C Bongioanni P Cova E Guareschi S Metelli MR. TNF and sTNFR1/2 plasma levels in ALS patients. J Neuroimmunol. (2008) 194:123–31. 10.1016/j.jneuroim.2007.10.028 18083240

[126] Blasco H Garcon G Patin F Veyrat-Durebex C Boyer J Devos D. Panel of oxidative stress and inflammatory biomarkers in ALS: a Pilot Study. Can J Neurol Sci. (2017) 44:90–5. 10.1017/cjn.2016.284 27774920

[127] Babu GN Kumar A Chandra R Puri SK Kalita J Misra UK. Elevated inflammatory markers in a group of amyotrophic lateral sclerosis patients from northern India. Neurochem Res. (2008) 33:1145–9. 10.1007/s11064-007-9564-x 18246426

[128] Hu Y Cao C Qin X-Y Yu Y Yuan J Zhao Y. Increased peripheral blood inflammatory cytokine levels in amyotrophic lateral sclerosis: a meta-analysis study. Sci Rep. (2017) 7:9094. 10.1038/s41598-017-09097-1 28831083 PMC5567306

[129] Goldknopf IL Sheta EA Bryson J Folsom B Wilson C Duty J. Complement C3c and related protein biomarkers in amyotrophic lateral sclerosis and Parkinson's disease. Biochem Biophys Res Commun. (2006) 342:1034–9. 10.1016/j.bbrc.2006.02.051 16516157

[130] Xu Z Lee A Nouwens A David Henderson R Ann Mccombe P. Mass spectrometry analysis of plasma from amyotrophic lateral sclerosis and control subjects. Amyotroph Lateral Scler Frontotemporal Degener. (2018) 19:362–76. 10.1080/21678421.2018.1433689 29384411

[131] Mantovani S Gordon R Macmaw JK Pfluger CMM Henderson RD Noakes PG. Elevation of the terminal complement activation products C5a and C5b-9 in ALS patient blood. J Neuroimmunol. (2014) 276:213–8. 10.1016/j.jneuroim.2014.09.005 25262158

[132] Hou L Jiao B Xiao T Zhou L Zhou Z Du J. Screening of SOD1, FUS and TARDBP genes in patients with amyotrophic lateral sclerosis in central-southern China. Sci Rep. (2016) 6:32478. 10.1038/srep32478 27604643 PMC5015023

[133] Nagel G Peter RS Rosenbohm A Koenig W Dupuis L Rothenbacher D. Adipokines, C-reactive protein and amyotrophic lateral sclerosis - results from a population- based ALS registry in Germany. Sci Rep. (2017) 7:4374. 10.1038/s41598-017-04706-5 28663573 PMC5491500

[134] Lunetta C Lizio A Maestri E Sansone VA Mora G Miller RG. Serum C-reactive protein as a prognostic biomarker in amyotrophic lateral sclerosis. JAMA Neurol. (2017) 74:660–7. 10.1001/jamaneurol.2016.6179 28384752 PMC5822215

[135] Pagliardini V Pagliardini S Corrado L Lucenti A Panigati L Bersano E. Chitotriosidase and lysosomal enzymes as potential biomarkers of disease progression in amyotrophic lateral sclerosis: a survey clinic-based study. J Neurol Sci. (2015) 348:245–50. 10.1016/j.jns.2014.12.016 25563799

[136] Patin F Corcia P Madji Hounoum B Veyrat-Durebex C Respaud E Piver E. Biological follow-up in amyotrophic lateral sclerosis: decrease in creatinine levels and increase in ferritin levels predict poor prognosis. Eur J Neurol. (2015) 22:1385–90. 10.1111/ene.12754 26095828

[137] Chiò A Calvo A Bovio G Canosa A Bertuzzo D Galmozzi F. Amyotrophic lateral sclerosis outcome measures and the role of albumin and creatinine: a population-based study. JAMA Neurol. (2014) 71:1134–42. 10.1001/jamaneurol.2014.1129 25048026

[138] Bozik ME Mitsumoto H Brooks BR Rudnicki SA Moore DH Zhang B. A *post hoc* analysis of subgroup outcomes and creatinine in the phase III clinical trial (EMPOWER) of dexpramipexole in ALS. Amyotroph Lateral Scler Frontotemporal Degener. (2014) 15:406–13. 10.3109/21678421.2014.943672 25125035

[139] Ikeda K Hirayama T Takazawa T Kawabe K Iwasaki Y. Relationships between disease progression and serum levels of lipid, urate, creatinine and ferritin in Japanese patients with amyotrophic lateral sclerosis: a cross-sectional study. Intern Med. (2012) 51:1501–8. 10.2169/internalmedicine.51.7465 22728481

[140] Rafiq MK Lee E Bradburn M McDermott CJ Shaw PJ. Creatine kinase enzyme level correlates positively with serum creatinine and lean body mass, and is a prognostic factor for survival in amyotrophic lateral sclerosis. Eur J Neurol. (2016) 23:1071–8. 10.1111/ene.12995 27029589

[141] van Eijk RPA Eijkemans MJC Ferguson TA Nikolakopoulos S Veldink JH van den Berg LH Monitoring disease progression with plasma creatinine in amyotrophic lateral sclerosis clinical trials. J Neurol Neurosurg Psychiatry. (2017) 89:156–61. 10.1136/jnnp-2017-317077 29084868 PMC5800333

[142] Tai H Cui L Guan Y Liu M Li X Shen D. Correlation of creatine kinase levels with clinical features and survival in amyotrophic lateral sclerosis. Front Neurol. (2017) 8:322. 10.3389/fneur.2017.00322 28717355 PMC5494475

[143] Ong ML Tan PF Holbrook JD. Predicting functional decline and survival in amyotrophic lateral sclerosis. PLoS ONE. (2017) 12:e0174925. 10.1371/journal.pone.0174925 28406915 PMC5390993

[144] De Felice B Guida M Guida M Coppola C De Mieri G Cotrufo R A miRNA signature in leukocytes from sporadic amyotrophic lateral sclerosis. Gene. (2012) 508:35–40. 10.1016/j.gene.2012.07.058 22903028

[145] Freischmidt A Muller K Zondler L Weydt P Volk AE Bozic AL. Serum microRNAs in patients with genetic amyotrophic lateral sclerosis and pre-manifest mutation carriers. Brain. (2014) 137(Pt. 11):2938–50. 10.1093/brain/awu249 25193138

[146] Freischmidt A Muller K Zondler L Weydt P Mayer B von Arnim CAF. Serum microRNAs in sporadic amyotrophic lateral sclerosis. Neurobiol Aging. (2015) 36:e15–20. 10.1016/j.neurobiolaging.2015.06.003 26142125

[147] Toivonen JM Manzano R Olivan S Zaragoza P Garcia-Redondo A Osta R. MicroRNA-206: a potential circulating biomarker candidate for amyotrophic lateral sclerosis. PLoS ONE. (2014) 9:e89065. 10.1371/journal.pone.0089065 24586506 PMC3930686

[148] Waller R Goodall EF Milo M Cooper-Knock J Da Costa M Hobson E. Serum miRNAs miR-206, 143-3p and 374b-5p as potential biomarkers for amyotrophic lateral sclerosis (ALS). Neurobiol Aging. (2017) 55:123–31. 10.1016/j.neurobiolaging.2017.03.027 28454844 PMC5455071

[149] Raheja R Regev K Healy BC Mazzola MA Beynon V Glehn FV. Correlating serum microRNAs and clinical parameters in Amyotrophic lateral sclerosis. Muscle Nerve. (2018) 58:261–9. 10.1002/mus.26106 29466830 PMC6103911

[150] de Andrade HM de Albuquerque M Avansini SH de SRC Dogini DB Nucci A MicroRNAs-424 and 206 are potential prognostic markers in spinal onset amyotrophic lateral sclerosis. J Neurol Sci. (2016) 368:19–24. 10.1016/j.jns.2016.06.046 27538595

[151] Takahashi I Hama Y Matsushima M Hirotani M Kano T Hohzen H. Identification of plasma microRNAs as a biomarker of sporadic amyotrophic lateral sclerosis. Mol Brain. (2015) 8:67. 10.1186/s13041-015-0161-7 26497046 PMC4619470

[152] Mariosa D Hammar N Malmstrom H Ingre C Junger I Ye W. Blood biomarkers of carbohydrate, lipid and apolipoprotein metabolisms and risk of amyotrophic lateral sclerosis: a more than 20 year follow-up of the Swedish AMORIS cohort. Ann Neurol. (2017) 81:718–28. 10.1002/ana.24936 28437840

[153] Cecchi M Messina P Airoldi L Pupillo E Bandettini di Poggio M Calvo A. (2014). Plasma amino acids patterns and age of onset of amyotrophic lateral sclerosis. Amyotroph Lateral Scler Frontotemporal Degener. 15:371–5. 10.3109/21678421.2014.920032 24904978

[154] Lawton KA Brown MV Alexander D Li Z Wulff JE Lawson R. Plasma metabolomic biomarker panel to distinguish patients with amyotrophic lateral sclerosis from disease mimics. Amyotroph Lateral Scler Frontotemporal Degener. (2014) 15:362–70. 10.3109/21678421.2014.908311 24984169

[155] Andreadou E Kapaki E Kokotis P Paraskevas GP Katsaros N Libitaki G. Plasma glutamate and glycine levels in patients with amyotrophic lateral sclerosis. In Vivo. (2008) 22:137–41. Available online at: http://iv.iiarjournals.org/content/22/1/137.long 18396796

[156] Andreadou E Kapaki E Kokotis P Paraskevas GP Katsaros N Libitaki G. Plasma glutamate and glycine levels in patients with amyotrophic lateral sclerosis: the effect of riluzole treatment. Clin Neurol Neurosurg. (2008) 110:222–6. 10.1016/j.clineuro.2007.10.018 18055102

[157] Niebroj-Dobosz I Janik P Kwiecinski H. Effect of Riluzole on serum amino acids in patients with amyotrophic lateral sclerosis. Acta Neurol Scand. (2002) 106:39–43. 10.1034/j.1600-0404.2002.00206.x 12067327

[158] Palma AS De Carvalho M Grammel N Pinto S Barata N Conradt HS. Proteomic analysis of plasma from Portuguese patients with familial amyotrophic lateral sclerosis. Amyotroph Lateral Scler. (2008) 9:339–49. 10.1080/17482960801934239 18608108

[159] Lawton KA Cudkowicz ME Brown MV Alexander D Caffrey R Wulff JE. Biochemical alterations associated with ALS. Amyotroph Lateral Scler. (2012) 13:110–8. 10.3109/17482968.2011.619197 22117131

[160] Conraux L Pech C Guerraoui H Loyaux D Ferrara P Guillemot JC. Plasma peptide biomarker discovery for amyotrophic lateral sclerosis by MALDI-TOF mass spectrometry profiling. PLoS ONE. (2013) 8:e79733. 10.1371/journal.pone.0079733 24224000 PMC3818176

[161] Wuolikainen A Jonsson P Ahnlund M Antti H Marklund SL Moritz T. Multi-platform mass spectrometry analysis of the CSF and plasma metabolomes of rigorously matched amyotrophic lateral sclerosis, Parkinson's disease and control subjects. Mol BioSyst. (2016) 12:1287–98. 10.1039/C5MB00711A 26883206

[162] Nadjar Y Gordon P Corcia P Bensimon G Pieroni L Meininger V. Elevated serum ferritin is associated with reduced survival in amyotrophic lateral sclerosis. PLoS ONE. (2012) 7:e45034. 10.1371/journal.pone.0045034 23024788 PMC3443244

[163] Keizman D Ish-Shalom M Berliner S Maimon N Vered Y Artamonov I. Low uric acid levels in serum of patients with ALS: further evidence for oxidative stress? J Neurol Sci. (2009) 285:95–9. 10.1016/j.jns.2009.06.002 19552925

[164] Oh SI Baek S Park JS Piao L Oh KW Kim SH. Prognostic role of serum levels of uric acid in amyotrophic lateral sclerosis. J Clin Neurol. (2015) 11:376–82. 10.3988/jcn.2015.11.4.376 26424237 PMC4596112

[165] Zheng Z Guo X Wei Q Song W Cao B Huang R Serum uric acid level is associated with the prevalence but not with survival of amyotrophic lateral sclerosis in a Chinese population. Metabol Brain Dis. (2014) 29:771–5. 10.1007/s11011-014-9510-y 24577631

[166] Zoccolella S Simone IL Capozzo R Tortelli R Leo A D'Errico E. An exploratory study of serum urate levels in patients with amyotrophic lateral sclerosis. J Neurol. (2011) 258:238–43. 10.1007/s00415-010-5735-9 20842370

[167] O'Reilly ÉJ Bjornevik K Schwarzschild MA McCullough ML Kolonel LN Le Marchand L. Pre-diagnostic plasma urate and the risk of amyotrophic lateral sclerosis. Amyotroph Lateral Scler Frontotemporal Degener. (2017) 19:194–200. 10.1080/21678421.2017.1418005 29277115 PMC6423442

[168] Paganoni S Zhang M Zárate AQ Jaffa M Yu H Cudkowicz ME. Uric acid levels predict survival in men with amyotrophic lateral sclerosis. J Neurol. (2012) 259:1923–8. 10.1007/s00415-012-6440-7 22323210 PMC4441749

[169] Paganoni S Nicholson K Chan J Shui A Schoenfeld D Sherman A. Urate levels predict survival in amyotrophic lateral sclerosis: analysis of the expanded Pooled Resource Open-Access ALS clinical trials database. Muscle Nerve. (2018) 57:430–4. 10.1002/mus.25950 28857199 PMC5812805

[170] Bocca B Forte G Oggiano R Clemente S Asara Y Peruzzu A. Level of neurotoxic metals in amyotrophic lateral sclerosis: a population-based case-control study. J Neurol Sci. (2015) 359:11–7. 10.1016/j.jns.2015.10.023 26671079

[171] Forte G Bocca B Oggiano R Clemente S Asara Y Sotgiu MA. Essential trace elements in amyotrophic lateral sclerosis (ALS): results in a population of a risk area of Italy. Neurol Sci. (2017) 38:1609–15. 10.1007/s10072-017-3018-2 28601974

[172] Oggiano R Solinas G Forte G Bocca B Farace C Pisano A. Trace elements in ALS patients and their relationships with clinical severity. Chemosphere. (2018) 197:457–66. 10.1016/j.chemosphere.2018.01.076 29366958

[173] Pratico D Lawson JA FitzGerald GA. Cyclooxygenase-dependent formation of the isoprostane. 8-epi prostaglandin F2 alpha. J Biol Chem. (1995) 270:9800–8. 10.1074/jbc.270.17.9800 7730359

[174] Ono S Imai T Matsubara S Takahashi K Jinnai K Yamano T. Decreased urinary concentrations of type IV collagen in amyotrophic lateral sclerosis. Acta Neurol Scand. (1999) 100:111–6. 10.1111/j.1600-0404.1999.tb01048.x 10442453

[175] Ono S Shimizu N Imai T Rodriguez GP. Urinary collagen metabolite excretion in amyotrophic lateral sclerosis. Muscle Nerve. (2001) 24:821–5. 10.1002/mus.1075 11360267

[176] Shepheard SR Chataway T Schultz DW Rush RA Rogers ML. The extracellular domain of neurotrophin receptor p75 as a candidate biomarker for amyotrophic lateral sclerosis. PLoS ONE. (2014) 9:e87398. 10.1371/journal.pone.0087398 24475283 PMC3903651

[177] Jia R Shepheard S Jin J Hu F Zhao X Xue L. Urinary extracellular domain of neurotrophin receptor p75 as a biomarker for amyotrophic lateral sclerosis in a Chinese cohort. Sci Rep. (2017) 7:5127. 10.1038/s41598-017-05430-w 28698670 PMC5506052

[178] Shepheard SR Wuu J Cardoso M Wiklendt L Dinning PG Chataway T Urinary p75(ECD): a prognostic, disease progression, and pharmacodynamic biomarker in ALS. Neurology. (2017) 88:1137–43. 10.1212/WNL.0000000000003741 28228570 PMC5373786

[179] Wilkinson ID Graves MJ Chapter 5: magnetic resonance imaging, In: Adam A Dixon AK Gillard JH Schaefer-Prokop CM editors, Grainger & Allison's Diagnostic Radiology: A Textbook of Medical Imaging, 6th ed., Philadelphia, PA: Churchill Livingstone, Elsevere (2014). p. 90–114.

[180] Sarchielli P Pelliccioli GP Tarducci R Chiarini P Presciutti O Gobbi G. Magnetic resonance imaging and 1H-magnetic resonance spectroscopy in amyotrophic lateral sclerosis. Neuroradiology. (2001) 43:189–97. 10.1007/s002340000472 11305749

[181] Grosskreutz J Kaufmann J Fradrich J Dengler R Heinze HJ Peschel T. Widespread sensorimotor and frontal cortical atrophy in Amyotrophic Lateral Sclerosis. BMC Neurol. (2006) 6:17. 10.1186/1471-2377-6-17 16638121 PMC1459868

[182] Mezzapesa DM D'Errico E Tortelli R Distaso E Cortese R Tursi M. Cortical thinning and clinical heterogeneity in amyotrophic lateral sclerosis. PLoS ONE. (2013) 8:e80748. 10.1371/journal.pone.0080748 24278317 PMC3835750

[183] Chuang K-S Tzeng H-L Chen S Wu J Chen T-J. Fuzzy c-means clustering with spatial information for image segmentation. Comput Med Imaging Graph. (2006) 30:9–15. 10.1016/j.compmedimag.2005.10.001 16361080

[184] Zhang Q Mao C Jin J Niu C Bai L Dang J. Side of limb-onset predicts laterality of gray matter loss in amyotrophic lateral sclerosis. Biomed Res Int. (2014) 2014:473250. 10.1155/2014/473250 25093168 PMC4100370

[185] Mezzapesa DM Ceccarelli A Dicuonzo F Carella A De Caro MF Lopez M. Whole-brain and regional brain atrophy in amyotrophic lateral sclerosis. Am J Neuroradiol. (2007) 28:255–9. Available online at: http://www.ajnr.org/content/28/2/255.long 17296989 PMC7977419

[186] Westeneng HJ Verstraete E Walhout R Schmidt R Hendrikse J Veldink JH. Subcortical structures in amyotrophic lateral sclerosis. Neurobiol Aging. (2015) 36:1075–82. 10.1016/j.neurobiolaging.2014.09.002 25281019

[187] Pinkhardt EH van Elst LT Ludolph AC Kassubek J. Amygdala size in amyotrophic lateral sclerosis without dementia: an *in vivo* study using MRI volumetry. BMC Neurol. (2006) 6:48. 10.1186/1471-2377-6-48 17189609 PMC1764753

[188] Thivard L Pradat PF Lehéricy S Lacomblez L Dormont D Chiras J. Diffusion tensor imaging and voxel based morphometry study in amyotrophic lateral sclerosis: relationships with motor disability. J Neurol Neurosurg Psychiatry. (2007) 78:889–92. 10.1136/jnnp.2006.101758 17635981 PMC2117724

[189] de Albuquerque M Branco LM Rezende TJ de Andrade HM Nucci A Franca MC Jr Longitudinal evaluation of cerebral and spinal cord damage in amyotrophic lateral sclerosis. Neuroimage Clin. (2017) 14:269–76. 10.1016/j.nicl.2017.01.024 28203530 PMC5294732

[190] Grolez G Kyheng M Lopes R Moreau C Timmerman K Auger F. MRI of the cervical spinal cord predicts respiratory dysfunction in ALS. Sci Rep. (2018) 8:1828. 10.1038/s41598-018-19938-2 29379040 PMC5789036

[191] Walhout R Westeneng HJ Verstraete E Hendrikse J Veldink JH van den Heuvel MP. Cortical thickness in ALS: towards a marker for upper motor neuron involvement. J Neurol Neurosurg Psychiatry. (2015) 86:288–94. 10.1136/jnnp-2013-306839 25121571

[192] Abrahams S Goldstein LH Suckling J Ng V Simmons A Chitnis X. Frontotemporal white matter changes in amyotrophic lateral sclerosis. J Neurol. (2005) 252:321–31. 10.1007/s00415-005-0646-x 15739047

[193] Crespi C Dodich A Cappa SF Canessa N Iannaccone S Corbo M. Multimodal MRI quantification of the common neurostructural bases within the FTD-ALS continuum. Neurobiol Aging. (2018) 62:95–104. 10.1016/j.neurobiolaging.2017.09.019 29131982

[194] Ellis CM Suckling J Amaro E Jr Bullmore ET Simmons A Williams SC. Volumetric analysis reveals corticospinal tract degeneration and extramotor involvement in ALS. Neurology. (2001) 57:1571–8. 10.1212/WNL.57.9.1571 11706094

[195] Kassubek J Unrath A Huppertz HJ Lule D Ethofer T Sperfeld AD. Global brain atrophy and corticospinal tract alterations in ALS, as investigated by voxel-based morphometry of 3-D MRI. Amyotroph Lateral Scler Other Motor Neuron Disord. (2005) 6:213–20. 10.1080/14660820510038538 16319024

[196] Meadowcroft MD Mutic NJ Bigler DC Wang JL Simmons Z Connor JR. Histological-MRI correlation in the primary motor cortex of patients with amyotrophic lateral sclerosis. J Magn Reson Imaging. (2015) 41:665–75. 10.1002/jmri.24582 24615949 PMC4145061

[197] Chen J Kostenko V Pioro EP Trapp BD. MR imaging-based estimation of upper motor neuron density in patients with amyotrophic lateral sclerosis: a feasibility study. Radiology. (2018) 287:955–64. 10.1148/radiol.2018162967 29361242 PMC5978454

[198] Filippi M Agosta F Abrahams S Fazekas F Grosskreutz J Kalra S. EFNS guidelines on the use of neuroimaging in the management of motor neuron diseases. Eur J Neurol. (2010) 17:526-e20. 10.1111/j.1468-1331.2010.02951.x 20136647 PMC3154636

[199] Charil A Corbo M Filippi M Kesavadas C Agosta F Munerati E. Structural and metabolic changes in the brain of patients with upper motor neuron disorders: a multiparametric MRI study. Amyotroph Lateral Scler. (2009) 10:269–79. 10.3109/17482960902777339 19922113

[200] Hecht MJ Fellner F Fellner C Hilz MJ Heuss D Neundorfer B. MRI-FLAIR images of the head show corticospinal tract alterations in ALS patients more frequently than T2-, T1- and proton-density-weighted images. J Neurol Sci. (2001) 186:37–44. 10.1016/S0022-510X(01)00503-2 11412870

[201] Peretti-Viton P Azulay JP Trefouret S Brunel H Daniel C Viton JM. MRI of the intracranial corticospinal tracts in amyotrophic and primary lateral sclerosis. Neuroradiology. (1999) 41:744–9. 10.1007/s002340050836 10552025

[202] Abe K Fujimura H Kobayashi Y Fujita N Yanagihara T. Degeneration of the pyramidal tracts in patients with amyotrophic lateral sclerosis. A premortem and postmortem magnetic resonance imaging study. J Neuroimaging. (1997) 7:208–12. 10.1111/jon199774208 9344001

[203] Mirowitz S Sartor K Gado M Torack R. Focal signal-intensity variations in the posterior internal capsule: normal MR findings and distinction from pathologic findings. Radiology. (1989) 172:535–9. 10.1148/radiology.172.2.2748836 2748836

[204] Oba H Araki T Ohtomo K Monzawa S Uchiyama G Koizumi K. Amyotrophic lateral sclerosis: T2 shortening in motor cortex at MR imaging. Radiology. (1993) 189:843–6. 10.1148/radiology.189.3.8234713 8234713

[205] Ignjatovic A Stevic Z Lavrnic S Dakovic M Bacic G. Brain iron MRI: a biomarker for amyotrophic lateral sclerosis. J Magn Reson Imaging. (2013) 38:1472–9. 10.1002/jmri.24121 23564606

[206] Adachi Y Sato N Saito Y Kimura Y Nakata Y Ito K. Usefulness of SWI for the detection of iron in the motor cortex in amyotrophic lateral sclerosis. J Neuroimaging. (2015) 25:443–51. 10.1111/jon.12127 24888543

[207] Kwan JY Jeong SY Van Gelderen P Deng HX Quezado MM Danielian LE. Iron accumulation in deep cortical layers accounts for MRI signal abnormalities in ALS: correlating 7 tesla MRI and pathology. PLoS ONE. (2012) 7:e35241. 10.1371/journal.pone.0035241 22529995 PMC3328441

[208] Hecht MJ Fellner C Schmid A Neundorfer B Fellner FA Cortical T2 signal shortening in amyotrophic lateral sclerosis is not due to iron deposits. Neuroradiology. (2005) 47:805–8. 10.1007/s00234-005-1421-5 16175348

[209] Vazquez-Costa JF Mazon M Carreres-Polo J Hervas D Perez-Tur J Marti-Bonmati L. Brain signal intensity changes as biomarkers in amyotrophic lateral sclerosis. Acta Neurol Scand. (2018) 137:262–71. 10.1111/ane.12863 29082510

[210] Endo H Sekiguchi K Shimada H Ueda T Kowa H Kanda F. Low signal intensity in motor cortex on susceptibility-weighted MR imaging is correlated with clinical signs of amyotrophic lateral sclerosis: a pilot study. J Neurol. (2018) 265:552–61. 10.1007/s00415-017-8728-0 29356968

[211] Ellis CM Simmons A Jones DK Bland J Dawson JM Horsfield MA. Diffusion tensor MRI assesses corticospinal tract damage in ALS. Neurology. (1999) 53:1051–8. 10.1212/WNL.53.5.1051 10496265

[212] Graham JM Papadakis N Evans J Widjaja E Romanowski CA Paley MN. Diffusion tensor imaging for the assessment of upper motor neuron integrity in ALS. Neurology. (2004) 63:2111–9. 10.1212/01.WNL.0000145766.03057.E7 15596758

[213] Filippini N Douaud G Mackay CE Knight S Talbot K Turner MR. Corpus callosum involvement is a consistent feature of amyotrophic lateral sclerosis. Neurology. (2010) 75:1645–52. 10.1212/WNL.0b013e3181fb84d1 21041787 PMC2974368

[214] Tang M Chen X Zhou Q Liu B Liu Y Liu S. Quantitative assessment of amyotrophic lateral sclerosis with diffusion tensor imaging in 3.0T magnetic resonance. Int J Clin Exp Med. (2015) 8:8295–303. Available online at: http://www.ijcem.com 26221413 PMC4509358

[215] Wang S Poptani H Woo JH Desiderio LM Elman LB McCluskey LF. Amyotrophic lateral sclerosis: diffusion-tensor and chemical shift MR imaging at 3.0 T. Radiology. (2006) 239:831–8. 10.1148/radiol.2393050573 16641339

[216] Iwata NK Aoki S Okabe S Arai N Terao Y Kwak S. Evaluation of corticospinal tracts in ALS with diffusion tensor MRI and brainstem stimulation. Neurology. (2008) 70:528–32. 10.1212/01.wnl.0000299186.72374.19 18268244

[217] Ciccarelli O Behrens TE Johansen-Berg H Talbot K Orrell RW Howard RS. Investigation of white matter pathology in ALS and PLS using tract-based spatial statistics. Hum Brain Mapping. (2009) 30:615–24. 10.1002/hbm.20527 18172851 PMC6870826

[218] Metwalli NS Benatar M Nair G Usher S Hu X Carew JD. Utility of axial and radial diffusivity from diffusion tensor MRI as markers of neurodegeneration in amyotrophic lateral sclerosis. Brain Res. (2010) 1348:156–64. 10.1016/j.brainres.2010.05.067 20513367

[219] Valsasina P Agosta F Benedetti B Caputo D Perini M Salvi F. Diffusion anisotropy of the cervical cord is strictly associated with disability in amyotrophic lateral sclerosis. J Neurol Neurosurg Psychiatry. (2007) 78:480–4. 10.1136/jnnp.2006.100032 17030586 PMC2117814

[220] Nair G Carew JD Usher S Lu D Hu XP Benatar M. Diffusion tensor imaging reveals regional differences in the cervical spinal cord in amyotrophic lateral sclerosis. Neuroimage. (2010) 53:576–83. 10.1016/j.neuroimage.2010.06.060 20600964

[221] Agosta F Pagani E Rocca MA Caputo D Perini M Salvi F. Voxel-based morphometry study of brain volumetry and diffusivity in amyotrophic lateral sclerosis patients with mild disability. Hum Brain Mapping. (2007) 28:1430–8. 10.1002/hbm.20364 17370339 PMC6871473

[222] Sage CA Van Hecke W Peeters R Sijbers J Robberecht W Parizel P. Quantitative diffusion tensor imaging in amyotrophic lateral sclerosis: revisited. Hum Brain Mapping. (2009) 30:3657–75. 10.1002/hbm.20794 19404990 PMC6870610

[223] van der Graaff MM Sage CA Caan MW Akkerman EM Lavini C Majoie CB Upper and extra-Moto neuron involvement in early Moto neuron disease: a diffusion tensor imaging study. Brain. (2011) 134(Pt. 4):1211–28. 10.1093/brain/awr016 21362631

[224] Baldaranov D Khomenko A Kobor I Bogdahn U Gorges M Kassubek J. Longitudinal diffusion tensor imaging-based assessment of tract alterations: an application to amyotrophic lateral sclerosis. Front Hum Neurosci. (2017) 11:567. 10.3389/fnhum.2017.00567 29259550 PMC5723297

[225] Foerster BR Dwamena BA Petrou M Carlos RC Callaghan BC Churchill CL. Diagnostic accuracy of diffusion tensor imaging in amyotrophic lateral sclerosis: a systematic review and individual patient data meta-analysis. Acad Radiol. (2013) 20:1099–106. 10.1016/j.acra.2013.03.017 23931423 PMC4384461

[226] Kassubek J Muller HP Del Tredici K Lule D Gorges M Braak H. Imaging the pathoanatomy of amyotrophic lateral sclerosis *in vivo*: targeting a propagation-based biological marker. J Neurol Neurosurg Psychiatry. (2018) 89:374–81. 10.1136/jnnp-2017-316365 29101254 PMC5869447

[227] Schuster C Hardiman O Bede P. Development of an automated MRI-based diagnostic protocol for amyotrophic lateral sclerosis using disease-specific pathognomonic features: a quantitative disease-state classification study. PLoS ONE. (2016) 11:e0167331. 10.1371/journal.pone.0167331 27907080 PMC5132189

[228] Ferraro PM Agosta F Riva N Copetti M Spinelli EG Falzone Y. Multimodal structural MRI in the diagnosis of motor neuron diseases. Neuroimage Clin. (2017) 16:240–7. 10.1016/j.nicl.2017.08.002 28794983 PMC5545829

[229] Kato Y Matsumura K Kinosada Y Narita Y Kuzuhara S Nakagawa T. Detection of pyramidal tract lesions in amyotrophic lateral sclerosis with magnetization-transfer measurements. Am J Neuroradiol. (1997) 18:1541–7. 9296197 PMC8338148

[230] Tanabe JL Vermathen M Miller R Gelinas D Weiner MW Rooney WD Reduced MTR in the corticospinal tract and normal T in Amyotrophic Lateral Sclerosis. Magn Reson Imaging. (1998) 16:1163–9. 10.1016/S0730-725X(98)00129-5 9858272 PMC2735261

[231] Cosottini M Pesaresi I Piazza S Diciotti S Belmonte G Battaglini M. Magnetization transfer imaging demonstrates a distributed pattern of microstructural changes of the cerebral cortex in amyotrophic lateral sclerosis. Am J Neuroradiol. (2011) 32:704–8. 10.3174/ajnr.A2356 21436337 PMC7965898

[232] Konrad C Henningsen H Bremer J Mock B Deppe M Buchinger C. Pattern of cortical reorganization in amyotrophic lateral sclerosis: a functional magnetic resonance imaging study. Exp Brain Res. (2002) 143:51–6. 10.1007/s00221-001-0981-9 11907690

[233] Schoenfeld MA Tempelmann C Gaul C Kuhnel GR Duzel E Hopf JM. Functional motor compensation in amyotrophic lateral sclerosis. J Neurol. (2005) 252:944–52. 10.1007/s00415-005-0787-y 15750701

[234] Konrad C Jansen A Henningsen H Sommer J Turski PA Brooks BR. Subcortical reorganization in amyotrophic lateral sclerosis. Exp Brain Res. (2006) 172:361–9. 10.1007/s00221-006-0352-7 16463149

[235] Stanton BR Williams VC Leigh PN Williams SC Blain CR Jarosz JM. Altered cortical activation during a motor task in ALS. Evidence for involvement of central pathways. J Neurol. (2007) 254:1260–7. 10.1007/s00415-006-0513-4 17385077

[236] Poujois A Schneider FC Faillenot I Camdessanche JP Vandenberghe N Thomas-Anterion C. Brain plasticity in the motor network is correlated with disease progression in amyotrophic lateral sclerosis. Hum Brain Mapping. (2013) 34:2391–401. 10.1002/hbm.22070 22461315 PMC6870334

[237] Mohammadi B Kollewe K Samii A Krampfl K Dengler R Munte TF Decreased brain activation to tongue movements in amyotrophic lateral sclerosis with bulbar involvement but not Kennedy syndrome. J Neurol. (2009b) 256:1263–9. 10.1007/s00415-009-5112-8 19353225

[238] Kollewe K Munte TF Samii A Dengler R Petri S Mohammadi B. Patterns of cortical activity differ in ALS patients with limb and/or bulbar involvement depending on motor tasks. J Neurol. (2011) 258:804–10. 10.1007/s00415-010-5842-7 21128080

[239] Lule D Diekmann V Kassubek J Kurt A Birbaumer N Ludolph AC. Cortical plasticity in amyotrophic lateral sclerosis: motor imagery and function. Neurorehabil Neural Repair. (2007) 21:518–26. 10.1177/1545968307300698 17476000

[240] Stanton BR Williams VC Leigh PN Williams SC Blain CR Giampietro VP. Cortical activation during motor imagery is reduced in amyotrophic lateral sclerosis. Brain Res. (2007) 1172:145–51. 10.1016/j.brainres.2007.07.044 17765211

[241] Mohammadi B Kollewe K Samii A Krampfl K Dengler R Munte TF. Changes of resting state brain networks in amyotrophic lateral sclerosis. Exp Neurol. (2009) 217:147–53. 10.1016/j.expneurol.2009.01.025 19416664

[242] Goldstein LH Newsom-Davis IC Bryant V Brammer M Leigh PN Simmons A. Altered patterns of cortical activation in ALS patients during attention and cognitive response inhibition tasks. J Neurol. (2011) 258:2186–98. 10.1007/s00415-011-6088-8 21556876 PMC3225607

[243] Lule D Diekmann V Anders S Kassubek J Kubler A Ludolph AC. Brain responses to emotional stimuli in patients with amyotrophic lateral sclerosis (ALS). J Neurol. (2007) 254:519–27. 10.1007/s00415-006-0409-3 17401515

[244] Palmieri A Naccarato M Abrahams S Bonato M D'Ascenzo C Balestreri S. Right hemisphere dysfunction and emotional processing in ALS: an fMRI study. J Neurol. (2010) 257:1970–8. 10.1007/s00415-010-5640-2 20593194

[245] Lule D Diekmann V Muller HP Kassubek J Ludolph AC Birbaumer N. Neuroimaging of multimodal sensory stimulation in amyotrophic lateral sclerosis. J Neurol Neurosurg Psychiatry. (2010) 81:899–906. 10.1136/jnnp.2009.192260 20543183

[246] Abrahams S Goldstein LH Simmons A Brammer M Williams SC Giampietro V. Word retrieval in amyotrophic lateral sclerosis: a functional magnetic resonance imaging study. Brain. (2004) 127(Pt. 7):1507–17. 10.1093/brain/awh170 15163610

[247] Ellis CM Simmons A Andrews C Dawson JM Williams SC Leigh PN. A proton magnetic resonance spectroscopic study in ALS: correlation with clinical findings. Neurology. (1998) 51:1104–9. 10.1212/WNL.51.4.1104 9781537

[248] Schuff N Rooney WD Miller R Gelinas DF Amend DL Maudsley AA. Reanalysis of multislice H MRSI in amyotrophic lateral sclerosis. Magn Reson Med. (2001) 45:513–6. 10.1002/1522-2594(200103)45:3<513::AID-MRM1067>3.0.CO;2-D 11241711

[249] Mitsumoto H Ulug AM Pullman SL Gooch CL Chan S Tang MX. Quantitative objective markers for upper and lower motor neuron dysfunction in ALS. Neurology. (2007) 68:1402–10. 10.1212/01.wnl.0000260065.57832.87 17452585

[250] Jones AP Gunawardena WJ Coutinho CM Gatt JA Shaw IC Mitchell JD. Preliminary results of proton magnetic resonance spectroscopy in motor neurone disease (amytrophic lateral sclerosis). J Neurol Sci. (1995) 129:85–9. 10.1016/0022-510X(95)00072-A 7595630

[251] Gredal O Rosenbaum S Topp S Karlsborg M Strange P Werdelin L. Quantification of brain metabolites in amyotrophic lateral sclerosis by localized proton magnetic resonance spectroscopy. Neurology. (1997) 48:878–81. 10.1212/WNL.48.4.878 9109871

[252] Han J Ma L. Study of the features of proton MR spectroscopy (H-MRS) on amyotrophic lateral sclerosis. J Magn Reson Imaging. (2010) 31:305–8. 10.1002/jmri.22053 20099342

[253] Sivak S Bittsansky M Kurca E Turcanova-Koprusakova M Grofik M Nosal V. Proton magnetic resonance spectroscopy in patients with early stages of amyotrophic lateral sclerosis. Neuroradiology. (2010) 52:1079–85. 10.1007/s00234-010-0685-6 20369234

[254] Foerster BR Callaghan BC Petrou M Edden RA Chenevert TL Feldman EL. Decreased motor cortex γ-aminobutyric acid in amyotrophic lateral sclerosis. Neurology. (2012) 78:1596–600. 10.1212/WNL.0b013e3182563b57 22517106 PMC3348851

[255] Foerster BR Pomper MG Callaghan BC Petrou M Edden RA Mohamed MA. An imbalance between excitatory and inhibitory neurotransmitters in amyotrophic lateral sclerosis revealed by use of 3-T proton magnetic resonance spectroscopy. JAMA Neurol. (2013) 70:1009–16. 10.1001/jamaneurol.2013.234 23797905 PMC4382938

[256] Weiduschat N Mao X Hupf J Armstrong N Kang G Lange DJ. Motor cortex glutathione deficit in ALS measured *in vivo* with the J-editing technique. Neurosci Lett. (2014) 570:102–7. 10.1016/j.neulet.2014.04.020 24769125

[257] Sako W Abe T Izumi Y Harada M Kaji R. The ratio of N-acetyl aspartate to glutamate correlates with disease duration of amyotrophic lateral sclerosis. J Clin Neurosci. (2016) 27:110–3. 10.1016/j.jocn.2015.08.044 26765768

[258] Cheong I Marjanska M Deelchand DK Eberly LE Walk D Oz G Ultra-High field proton MR spectroscopy in early-stage amyotrophic lateral sclerosis. Neurochem Res. (2017) 42:1833–44. 10.1007/s11064-017-2248-2 28367604 PMC5488866

[259] Atassi N Xu M Triantafyllou C Keil B Lawson R Cernasov P. Ultra high-field (7tesla) magnetic resonance spectroscopy in amyotrophic lateral sclerosis. PLoS ONE. (2017) 12:e0177680. 10.1371/journal.pone.0177680 28498852 PMC5428977

[260] Wang Y Li X Chen W Wang Z Xu Y Luo J. Detecting neuronal dysfunction of hand motor cortex in ALS: a MRSI study. Somatosens Mot Res. (2017) 34:15–20. 10.1080/08990220.2016.1275544 28114839

[261] Pioro EP. MR spectroscopy in amyotrophic lateral sclerosis/motor neuron disease. J Neurol Sci. (1997) 152(Suppl. 1):S49–53. 10.1016/S0022-510X(97)00244-X 9419054

[262] Cwik VA Hanstock CC Allen PS Martin WR. Estimation of brainstem neuronal loss in amyotrophic lateral sclerosis with *in vivo* proton magnetic resonance spectroscopy. Neurology. (1998) 50:72–7. 10.1212/WNL.50.1.72 9443460

[263] Pioro EP Majors AW Mitsumoto H Nelson DR Ng TC. 1H-MRS evidence of neurodegeneration and excess glutamate + glutamine in ALS medulla. Neurology. (1999) 53:71–9. 10.1212/WNL.53.1.71 10408539

[264] Carew JD Nair G Pineda-Alonso N Usher S Hu X Benatar M. Magnetic resonance spectroscopy of the cervical cord in amyotrophic lateral sclerosis. Amyotroph Lateral Scler. (2011) 12:185–91. 10.3109/17482968.2010.515223 21143004

[265] Ikeda K Murata K Kawase Y Kawabe K Kano O Yoshii Y. Relationship between cervical cord 1H-magnetic resonance spectroscopy and clinoco-electromyographic profile in amyotrophic lateral sclerosis. Muscle Nerve. (2013) 47:61–7. 10.1002/mus.23467 23042532

[266] Verma G Woo JH Chawla S Wang S Sheriff S Elman LB. Whole-brain analysis of amyotrophic lateral sclerosis by using echo-planar spectroscopic imaging. Radiology. (2013) 267:851–7. 10.1148/radiol.13121148 23360740 PMC3662903

[267] Pyra T Hui B Hanstock C Concha L Wong JC Beaulieu C. Combined structural and neurochemical evaluation of the corticospinal tract in amyotrophic lateral sclerosis. Amyotroph Lateral Scler. (2010) 11:157–65. 10.3109/17482960902756473 19242831

[268] Chan S Shungu DC Douglas-Akinwande A Lange DJ Rowland LP. Motor neuron diseases: comparison of single-voxel proton MR spectroscopy of the motor cortex with MR imaging of the brain. Radiology. (1999) 212:763–9. 10.1148/radiology.212.3.r99au35763 10478245

[269] Cervo A Cocozza S Saccà F Giorgio SM Morra VB Tedeschi E. The combined use of conventional MRI and MR spectroscopic imaging increases the diagnostic accuracy in amyotrophic lateral sclerosis. Eur J Radiol. (2015) 84:151–7. 10.1016/j.ejrad.2014.10.019 25466774

[270] Kaufmann P Pullman SL Shungu DC Chan S Hays AP Del Bene ML. Objective tests for upper motor neuron involvement in amyotrophic lateral sclerosis (ALS). Neurology. (2004) 62:1753–7. 10.1212/01.WNL.0000125182.17874.59 15159473

[271] Foerster BR Carlos RC Dwamena BA Callaghan BC Petrou M Edden RA. Multimodal MRI as a diagnostic biomarker for amyotrophic lateral sclerosis. Ann Clin Transl Neurol. (2014) 1:107–14. 10.1002/acn3.30 25356389 PMC4212480

[272] Unrath A Ludolph AC Kassubek J. Brain metabolites in definite amyotrophic lateral sclerosis. A longitudinal proton magnetic resonance spectroscopy study. J Neurol. (2007) 254:1099–106. 10.1007/s00415-006-0495-2 17431700

[273] Bowen BC Pattany PM Bradley WG Murdoch JB Rotta F Younis AA. MR imaging and localized proton spectroscopy of the precentral gyrus in amyotrophic lateral sclerosis. Am J Neuroradiol. (2000) 21:647–58. Available online at: http://www.ajnr.org/content/21/4/647.long 10782773 PMC7976640

[274] Rule RR Suhy J Schuff N Gelinas DF Miller RG Weiner MW. Reduced NAA in motor and non-motor brain regions in amyotrophic lateral sclerosis: a cross-sectional and longitudinal study. Amyotroph Lateral Scler Other Motor Neuron Disord. (2004) 5:141–9. 10.1080/14660820410017109 15512902 PMC2744639

[275] Kalra S Cashman NR Genge A Arnold DL. Recovery of N-acetylaspartate in corticomotor neurons of patients with ALS after riluzole therapy. Neuroreport. (1998) 9:1757–61. 10.1097/00001756-199806010-00016 9665596

[276] Kalra S Tai P Genge A Arnold DL. Rapid improvement in cortical neuronal integrity in amyotrophic lateral sclerosis detected by proton magnetic resonance spectroscopic imaging. J Neurol. (2006) 253:1060–3. 10.1007/s00415-006-0162-7 16609809

[277] Kalra S Cashman NR Caramanos Z Genge A Arnold DL. Gabapentin therapy for amyotrophic lateral sclerosis: lack of improvement in neuronal integrity shown by MR spectroscopy. Am J Neuroradiol. (2003) 24:476–80. Available online at: http://www.ajnr.org/content/24/3/476.long 12637300 PMC7973590

[278] Khiat A D'Amour M Souchon F Boulanger Y. MRS study of the effects of minocycline on markers of neuronal and microglial integrity in ALS. Magn Reson Imaging. (2010) 28:1456–60. 10.1016/j.mri.2010.06.032 20832222

[279] Atassi N Ratai EM Greenblatt DJ Pulley D Zhao Y Bombardier J. A phase I, pharmacokinetic, dosage escalation study of creatine monohydrate in subjects with amyotrophic lateral sclerosis. Amyotroph Lateral Scler. (2010) 11:508–13. 10.3109/17482961003797130 20698808 PMC3045755

[280] Sacca F Quarantelli M Rinaldi C Tucci T Piro R Perrotta G. A randomized controlled clinical trial of growth hormone in amyotrophic lateral sclerosis: clinical, neuroimaging, and hormonal results. J Neurol. (2012) 259:132–8. 10.1007/s00415-011-6146-2 21706151

[281] Garcia Santos JM Inuggi A Gomez Espuch J Vazquez C Iniesta F Blanquer M Spinal cord infusion of stem cells in amyotrophic lateral sclerosis: magnetic resonance spectroscopy shows metabolite improvement in the precentral gyrus. Cytotherapy. (2016) 18:785–96. 10.1016/j.jcyt.2016.03.296 27173751

[282] Govind V Sharma KR Maudsley AA Arheart KL Saigal G Sheriff S. Comprehensive evaluation of corticospinal tract metabolites in amyotrophic lateral sclerosis using whole-brain 1H MR spectroscopy. PLoS ONE. (2012) 7:e35607. 10.1371/journal.pone.0035607 22539984 PMC3335096

[283] Staff NP Amrami KK Howe BM MRI abnormalities of peripheral nerve and muscle are common in amyotrophic lateral sclerosis and share features with multifocal motor neuropathy. Muscle Nerve. (2015) 52:137–9. 10.1002/mus.24630 25736373 PMC4474748

[284] Cha CH Patten BM. Amyotrophic lateral sclerosis: abnormalities of the tongue on magnetic resonance imaging. Ann Neurol. (1989) 25:468–72. 10.1002/ana.410250508 2774487

[285] Simon NG Lagopoulos J Paling S Pfluger C Park SB Howells J. Peripheral nerve diffusion tensor imaging as a measure of disease progression in ALS. J Neurol. (2017) 264:882–90. 10.1007/s00415-017-8443-x 28265751

[286] Bryan WW Reisch JS McDonald G Herbelin LL Barohn RJ Fleckenstein JL. Magnetic resonance imaging of muscle in amyotrophic lateral sclerosis. Neurology. (1998) 51:110–3. 10.1212/WNL.51.1.110 9674787

[287] Gerevini S Agosta F Riva N Spinelli EG Pagani E Caliendo G. MR Imaging of brachial plexus and limb-girdle muscles in patients with amyotrophic lateral sclerosis. Radiology. (2016) 279:553–61. 10.1148/radiol.2015150559 26583760

[288] Jenkins TM Alix JJP David C Pearson E Rao DG Hoggard N. Imaging muscle as a potential biomarker of denervation in motor neuron disease. J Neurol Neurosurg Psychiatry. (2018) 89:248–55. 10.1136/jnnp-2017-316744 29089397 PMC5869448

[289] Ryan TE Erickson ML Verma A Chavez J Rivner MH McCully KK. Skeletal muscle oxidative capacity in amyotrophic lateral sclerosis. Muscle Nerve. (2014) 50:767–74. 10.1002/mus.24223 24616062

[290] Grehl T Fischer S Muller K Malin JP Zange J. A prospective study to evaluate the impact of 31P-MRS to determinate mitochondrial dysfunction in skeletal muscle of ALS patients. Amyotroph Lateral Scler. (2007) 8:4–8. 10.1080/17482960600765065 17364428

[291] Zochodne DW Thompson RT Driedger AA Strong MJ Gravelle D Bolton CF. Metabolic changes in human muscle denervation: topical 31P NMR spectroscopy studies. Magn Reson Med. (1988) 7:373–83. 10.1002/mrm.1910070402 3173055

[292] Sharma KR Kent-Braun JA Majumdar S Huang Y Mynhier M Weiner MW. Physiology of fatigue in amyotrophic lateral sclerosis. Neurology. (1995) 45:733–40. 10.1212/WNL.45.4.733 7723963

[293] Kent-Braun JA Miller RG. Central fatigue during isometric exercise in amyotrophic lateral sclerosis. Muscle Nerve. (2000) 23:909–14. 10.1002/(SICI)1097-4598(200006)23:6<909::AID-MUS10>3.0.CO;2-V 10842267

[294] Hatazawa J Brooks RA Dalakas MC Mansi L Di Chiro G. Cortical motor-sensory hypometabolism in amyotrophic lateral sclerosis: a PET study. J Comput Assist Tomogr. (1988) 12:630–6. 10.1097/00004728-198807000-00019 3260610

[295] Pagani M Chio A Valentini MC Oberg J Nobili F Calvo A. Functional pattern of brain FDG-PET in amyotrophic lateral sclerosis. Neurology. (2014) 83:1067–74. 10.1212/WNL.0000000000000792 25122207

[296] Canosa A Pagani M Cistaro A Montuschi A Iazzolino B Fania P. 18F-FDG-PET correlates of cognitive impairment in ALS. Neurology. (2016) 86:44–9. 10.1212/WNL.0000000000002242 26590270

[297] Van Weehaeghe D Ceccarini J Delva A Robberecht W Van Damme P Van Laere K Prospective validation of 18F-FDG brain PET discriminant analysis methods in the diagnosis of amyotrophic lateral sclerosis. J Nucl Med. (2016) 57:1238–43. 10.2967/jnumed.115.166272 26940764

[298] Cistaro A Valentini MC Chiò A Nobili F Calvo A Moglia C. Brain hypermetabolism in amyotrophic lateral sclerosis: a FDG PET study in ALS of spinal and bulbar onset. Eur J Nucl Med Mol Imaging. (2012) 39:251–9. 10.1007/s00259-011-1979-6 22089661

[299] D'hulst L Van Weehaeghe D Chiò A Calvo A Moglia C Canosa A Multicenter validation of [18F]-FDG PET and support-vector machine discriminant analysis in automatically classifying patients with amyotrophic lateral sclerosis versus controls. Amyotroph Lateral Scler Frontotemporal Degener. 10.1080/21678421.2018.1476548. [Epub ahead of print]. 29862846

[300] Turner MR Cagnin A Turkheimer FE Miller CC Shaw CE Brooks DJ Evidence of widespread cerebral microglial activation in amyotrophic lateral sclerosis: an [11C](R)-PK11195 positron emission tomography study. Neurobiol Dis. (2004) 15:601–9. 10.1016/j.nbd.2003.12.012 15056468

[301] Zurcher NR Loggia ML Lawson R Chonde DB Izquierdo-Garcia D Yasek JE Increased *in vivo* glial activation in patients with amyotrophic lateral sclerosis: assessed with [C]-PBR2(2016) 8. Neuroimage Clin. (2015) 7:409–14. 10.1016/j.nicl.2015.01.009 25685708 PMC4310932

[302] Alshikho MJ Zürcher NR Loggia ML Cernasov P Chonde DB Izquierdo Garcia D. Glial activation colocalizes with structural abnormalities in amyotrophic lateral sclerosis. Neurology. (2016) 87:2554–61. 10.1212/WNL.0000000000003427 27837005 PMC5207001

[303] Ratai E-M Alshikho MJ Zürcher NR Loggia ML Cebulla CL Cernasov P Integrated imaging of [C]-PBR28 PET, MR diffusion and magnetic resonance spectroscopy H-MRS in amyotrophic lateral sclerosis. NeuroImage Clin. (2018) 20:357–64. 10.1016/j.nicl.2018.08.007 30112276 PMC6092554

[304] Lloyd CM Richardson MP Brooks DJ Al-Chalabi A Leigh PN Extramotor involvement in ALS: PET studies with the GABAA ligand [11C]flumazenil. Brain. (2000) 123:2289–96. 10.1093/brain/123.11.2289 11050028

[305] Turner MR Hammers A Al-Chalabi A Shaw CE Andersen PM Brooks DJ. Distinct cerebral lesions in sporadic and ‘D90A’ SOD1 ALS: studies with [11C]flumazenil PET. Brain. (2005) 128:1323–9. 10.1093/brain/awh509 15843422

[306] Turner MR Rabiner EA Hammers A Al-Chalabi A Grasby PM Shaw CE. [11C]-WAY100635 PET demonstrates marked 5-HT1A receptor changes in sporadic ALS. Brain. (2005) 128(Pt. 4):896–905. 10.1093/brain/awh428 15689356

[307] Ikawa M Okazawa H Tsujikawa T Matsunaga A Yamamura O Mori T. Increased oxidative stress is related to disease severity in the ALS motor cortex. A PET study. Neurology. (2015) 84:2033–9. 10.1212/WNL.0000000000001588 25904686

[308] Turner MR Grosskreutz J Kassubek J Abrahams S Agosta F Benatar M. Towards a neuroimaging biomarker for amyotrophic lateral sclerosis. Lancet Neurol. (2011b) 10:400–3. 10.1016/S1474-4422(11)70049-7 21511189

[309] McComas AJ Fawcett PR Campbell MJ Sica RE. Electrophysiological estimation of the number of motor units within a human muscle. J Neurol Neurosurg Psychiatry. (1971) 34:121–31. 10.1136/jnnp.34.2.121 5571599 PMC493722

[310] Gooch CL Doherty TJ Chan KM Bromberg MB Lewis RA Stashuk DW. Motor unit number estimation: a technology and literature review. Muscle Nerve. (2014) 50:884–93. 10.1002/mus.24442 25186553

[311] Benmouna K Milants C Wang FC. Correlations between MUNIX and adapted multiple point stimulation MUNE methods. Clin Neurophysiol. (2017) 129:341–4. 10.1016/j.clinph.2017.11.012 29288988

[312] Kadrie HA Yates SK Milner-Brown HS Brown WF. Multiple point electrical stimulation of ulnar and median nerves. J Neurol Neurosurg Psychiatry. (1976) 39:973–85. 1003242 10.1136/jnnp.39.10.973 PMC492500

[313] Shefner JM Watson ML Simionescu L Caress JB Burns TM Maragakis NJ. Multipoint incremental motor unit number estimation as an outcome measure in ALS. Neurology. (2011) 77:235–41. 10.1212/WNL.0b013e318225aabf 21676915 PMC3136054

[314] Yuen EC Olney RK. Longitudinal study of fiber density and motor unit number estimate in patients with amyotrophic lateral sclerosis. Neurology. (1997) 49:573–8. 10.1212/WNL.49.2.573 9270599

[315] Ridall PG Pettitt AN Henderson RD McCombe PA. Motor unit number estimation–a Bayesian approach. Biometrics. (2006) 62:1235–50. 10.1111/j.1541-0420.2006.00577.x 17156299

[316] de Carvalho M Swash M Sensitivity of electrophysiological tests for upper and lower motor neuron dysfunction in ALS: a six-month longitudinal study. Muscle Nerve. (2010) 41:208–11. 19697379 10.1002/mus.21495

[317] Aggarwal A Nicholson G. Detection of preclinical motor neurone loss in SOD1 mutation carriers using motor unit number estimation. J Neurol Neurosurg Psychiatry. (2002) 73:199–201. 10.1136/jnnp.73.2.199 12122184 PMC1737965

[318] Shefner JM Cudkowicz ME Zhang H Schoenfeld D Jillapalli D. Revised statistical motor unit number estimation in the Celecoxib/ALS trial. Muscle Nerve. (2007) 35:228–34. 10.1002/mus.20671 17058270

[319] de Carvalho M Swash M Lower motor neuron dysfunction in ALS. Clin Neurophysiol. (2016) 127:2670–781. 10.1016/j.clinph.2016.03.024 27117334

[320] van Dijk JP Schelhaas HJ Van Schaik IN Janssen HM Stegeman DF Zwarts MJ Monitoring disease progression using high-density motor unit number estimation in amyotrophic lateral sclerosis. Muscle Nerve. (2010) 42:239–44. 10.1002/mus.21680 20544934

[321] Bostock H. Estimating motor unit numbers from a CMAP scan. Muscle Nerve. (2016) 53:889–96. 10.1002/mus.24945 26479267

[322] Jacobsen AB Bostock H Tankisi H Following disease progression in motor neuron disorders with 3 MUNE methods. Muscle Nerve. (2018) 59:82–7. 10.1002/mus.26304 30025164

[323] Nandedkar SD Nandedkar DS Barkhaus PE Stalberg EV. Motor unit number index (MUNIX). IEEE Trans Biomed Eng. (2004) 51:2209–11. 10.1109/TBME.2004.834281 15605872

[324] Neuwirth C Nandedkar S Stalberg E Barkhaus PE Carvalho M Furtula J. Motor Unit Number Index (MUNIX): a novel neurophysiological marker for neuromuscular disorders; test-retest reliability in healthy volunteers. Clin Neurophysiol. (2011) 122:1867–72. 10.1016/j.clinph.2011.02.017 21396884

[325] Neuwirth C Barkhaus PE Burkhardt C Castro J Czell D de Carvalho M. Tracking motor neuron loss in a set of six muscles in amyotrophic lateral sclerosis using the Motor Unit Number Index (MUNIX): a 15-month longitudinal multicentre trial. J Neurol Neurosurg Psychiatry. (2015) 86:1172–9. 10.1136/jnnp-2015-310509 25935892

[326] Jacobsen AB Bostock H Fuglsang-Frederiksen A Duez L Beniczky S Moller AT. Reproducibility, and sensitivity to motor unit loss in amyotrophic lateral sclerosis, of a novel MUNE method: MScanFit MUNE. Clin Neurophysiol. (2017) 128:1380–8. 10.1016/j.clinph.2017.03.045 28461135

[327] Boekestein WA Schelhaas HJ van Putten MJ Stegeman DF Zwarts MJ van Dijk JP. Motor unit number index (MUNIX) versus motor unit number estimation (MUNE): a direct comparison in a longitudinal study of ALS patients. Clin Neurophysiol. (2012) 123:1644–9. 10.1016/j.clinph.2012.01.004 22321299

[328] Neuwirth C Braun N Claeys KG Bucelli R Fournier C Bromberg M. Implementing Motor Unit Number Index (MUNIX) in a large clinical trial: real world experience from 27 centres. Clin Neurophysiol. (2018) 129:1756–62. 10.1016/j.clinph.2018.04.614 29803404

[329] Escorcio-Bezerra ML Abrahao A Nunes KF De Oliveira Braga NI Oliveira ASB Zinman L. Motor unit number index and neurophysiological index as candidate biomarkers of presymptomatic motor neuron loss in amyotrophic lateral sclerosis. Muscle Nerve. (2018) 58:204–12. 10.1002/mus.26087 29381812

[330] Neuwirth C Barkhaus PE Burkhardt C Castro J Czell D de Carvalho M. Motor Unit Number Index (MUNIX) detects motor neuron loss in pre-symptomatic muscles in Amyotrophic Lateral Sclerosis. Clin Neurophysiol. (2017) 128:495–500. 10.1016/j.clinph.2016.11.026 28043769

[331] Grimaldi S Duprat L Grapperon AM Verschueren A Delmont E Attarian S. Global motor unit number index sum score for assessing the loss of lower motor neurons in amyotrophic lateral sclerosis. Muscle Nerve. (2017) 56:202–6. 10.1002/mus.25595 28164325

[332] Escorcio-Bezerra ML Abrahao A de Castro I Chieia MAT de Azevedo LA Pinheiro DS. MUNIX: reproducibility and clinical correlations in amyotrophic lateral sclerosis. Clin Neurophysiol. (2016) 127:2979–84. 10.1016/j.clinph.2016.06.011 27458836

[333] de Carvalho M Swash M Nerve conduction studies in amyotrophic lateral sclerosis. Muscle Nerve. (2000) 23:344–52. 10.1002/(SICI)1097-4598(200003)23:3<344::AID-MUS5>3.0.CO;2-N 10679710

[334] Cheah BC Vucic S Krishnan AV Boland RA Kiernan MC. Neurophysiological index as a biomarker for ALS progression: validity of mixed effects models. Amyotroph Lateral Scler. (2011) 12:33–8. 10.3109/17482968.2010.531742 21271790

[335] de Carvalho M Scotto M Lopes A Swash M Quantitating progression in ALS. Neurology. (2005) 64:1783–5. 10.1212/01.WNL.0000162036.76024.AB 15911812

[336] Geevasinga N Menon P Nicholson GA Ng K Howells J Kril JJ. Cortical function in asymptomatic carriers and patients with C9orf72 amyotrophic lateral sclerosis. JAMA Neurol. (2015) 72:1268–74. 10.1001/jamaneurol.2015.1872 26348842 PMC4707047

[337] Vucic S Kiernan MC. Novel threshold tracking techniques suggest that cortical hyperexcitability is an early feature of motor neuron disease. Brain. (2006) 129(Pt. 9):2436–46. 10.1093/brain/awl172 16835248

[338] Park SB Kiernan MC Vucic S. Axonal excitability in amyotrophic lateral sclerosis: axonal excitability in ALS. Neurotherapeutics. (2017) 14:78–90. 10.1007/s13311-016-0492-9 27878516 PMC5233634

[339] Bostock H Cikurel K Burke D. Threshold tracking techniques in the study of human peripheral nerve. Muscle Nerve. (1998) 21:137–58. 9466589 10.1002/(sici)1097-4598(199802)21:2<137::aid-mus1>3.0.co;2-c

[340] Shibuta Y Shimatani Y Nodera H Izumi Y Kaji R. Increased variability of axonal excitability in amyotrophic lateral sclerosis. Clin Neurophysiol. (2013) 124:2046–53. 10.1016/j.clinph.2013.02.117 23726502

[341] Kanai K Kuwabara S Misawa S Tamura N Ogawara K Nakata M. Altered axonal excitability properties in amyotrophic lateral sclerosis: impaired potassium channel function related to disease stage. Brain. (2006) 129(Pt. 4):953–62. 10.1093/brain/awl024 16467388

[342] Vucic S Kiernan MC. Upregulation of persistent sodium conductances in familial ALS. J Neurol Neurosurg Psychiatry. (2010) 81:222–7. 10.1136/jnnp.2009.183079 19726402

[343] Kuo JJ Siddique T Fu R Heckman CJ. Increased persistent Na(+) current and its effect on excitability in motoneurones cultured from mutant SOD1 mice. J Physiol. (2005) 563(Pt. 3):843–54. 10.1113/jphysiol.2004.074138 15649979 PMC1665614

[344] Stys PK Waxman SG Ransom BR. Reverse operation of the Na(+)-Ca2+ exchanger mediates Ca2+ influx during anoxia in mammalian CNS white matter. Ann N Y Acad Sci. (1991) 639:328–32. 10.1111/j.1749-6632.1991.tb17321.x 1785859

[345] Geevasinga N Menon P Howells J Nicholson GA Kiernan MC Vucic S. Axonal ion channel dysfunction in c9orf72 familial amyotrophic lateral sclerosis. JAMA Neurol. (2015) 72:49–57. 10.1001/jamaneurol.2014.2940 25384182

[346] Kanai K Yokota T Shibuya K Nakazato T Kanouchi T Iwai Y Increased motor axonal persistent sodium currents is associated with rapid regional spreading in amyotrophic lateral sclerosis. J Neurol Sci. (2017) 381:558 10.1016/j.jns.2017.08.3779

[347] Kanai K Shibuya K Sato Y Misawa S Nasu S Sekiguchi Y. Motor axonal excitability properties are strong predictors for survival in amyotrophic lateral sclerosis. J Neurol Neurosurg Psychiatry. (2012) 83:734–8. 10.1136/jnnp-2011-301782 22566594

[348] Rutkove SB. Electrical impedance myography: background, current state, and future directions. Muscle Nerve. (2009) 40:936–46. 10.1002/mus.21362 19768754 PMC2824130

[349] Henderson RD McCombe PA. Assessment of motor units in neuromuscular disease. Neurotherapeutics. (2017) 14:69–77. 10.1007/s13311-016-0473-z 27600517 PMC5233620

[350] Zaidman CM Wang LL Connolly AM Florence J Wong BL Parsons JA. Electrical impedance myography in Duchenne muscular dystrophy and healthy controls: a multicenter study of reliability and validity. Muscle Nerve. (2015) 52:592–7. 10.1002/mus.24611 25702806

[351] Rutkove SB Lee KS Shiffman CA Aaron R. Test-retest reproducibility of 50 kHz linear-electrical impedance myography. Clin Neurophysiol. (2006) 117:1244–8. 10.1016/j.clinph.2005.12.029 16644269

[352] Rutkove SB Caress JB Cartwright MS Burns TM Warder J David WS. Electrical impedance myography correlates with standard measures of ALS severity. Muscle Nerve. (2014) 49:441–3. 10.1002/mus.24128 24273034

[353] Rutkove SB Caress JB Cartwright MS Burns TM Warder J David WS. Electrical impedance myography as a biomarker to assess ALS progression. Amyotroph Lateral Scler. (2012) 13:439–45. 10.3109/17482968.2012.688837 22670883 PMC3422377

[354] Garmirian LP Chin AB Rutkove SB. Discriminating neurogenic from myopathic disease via measurement of muscle anisotropy. Muscle Nerve. (2009) 39:16–24. 10.1002/mus.21115 19058193 PMC2719295

[355] Sanchez B Rutkove SB. Electrical impedance myography and its applications in neuromuscular disorders. Neurotherapeutics. (2017) 14:107–18. 10.1007/s13311-016-0491-x 27812921 PMC5233633

[356] Chio A Logroscino G Hardiman O Swingler R Mitchell D Beghi E. Prognostic factors in ALS: a critical review. Amyotroph Lateral Scler. (2009) 10:310–23. 10.3109/17482960802566824 19922118 PMC3515205

[357] McIlduff CE Yim SJ Pacheck AK Rutkove SB. Optimizing electrical impedance myography of the tongue in amyotrophic lateral sclerosis. Muscle Nerve. (2017) 55:539–43. 10.1002/mus.25375 27511962

[358] Shellikeri S Yunusova Y Green JR Pattee GL Berry JD Rutkove SB. Electrical impedance myography in the evaluation of the tongue musculature in amyotrophic lateral sclerosis. Muscle Nerve. (2015) 52:584–91. 10.1002/mus.24565 25580728 PMC4499330

[359] de Carvalho M Dengler R Eisen A England JD Kaji R Kimura J. Electrodiagnostic criteria for diagnosis of ALS. Clin Neurophysiol. (2008) 119:497–503. 10.1016/j.clinph.2007.09.143 18164242

[360] Vucic S Kiernan MC. Transcranial magnetic stimulation for the assessment of neurodegenerative disease. Neurotherapeutics. (2017) 14:91–106. 10.1007/s13311-016-0487-6 27830492 PMC5233629

[361] Barker AT Jalinous R Freeston IL. Non-invasive magnetic stimulation of human motor cortex. Lancet. (1985) 1:1106–7. 10.1016/S0140-6736(85)92413-4 2860322

[362] Vucic S Ziemann U Eisen A Hallett M Kiernan MC. Transcranial magnetic stimulation and amyotrophic lateral sclerosis: pathophysiological insights. J Neurol Neurosurg Psychiatry. (2013) 84:1161–70. 10.1136/jnnp-2012-304019 23264687 PMC3786661

[363] Vucic S Nicholson GA Kiernan MC Cortical hyperexcitability may precede the onset of familial amyotrophic lateral sclerosis. Brain. (2008) 131(Pt. 6):1540–50. 10.1093/brain/awn071 18469020

[364] Menon P Geevasinga N Yiannikas C Howells J Kiernan MC Vucic S. Sensitivity and specificity of threshold tracking transcranial magnetic stimulation for diagnosis of amyotrophic lateral sclerosis: a prospective study. Lancet Neurol. (2015) 14:478–84. 10.1016/S1474-4422(15)00014-9 25843898

[365] Menon P Geevasinga N van den Bos M Yiannikas C Kiernan MC Vucic S. Cortical hyperexcitability and disease spread in amyotrophic lateral sclerosis. Eur J Neurol. (2017) 24:816–24. 10.1111/ene.13295 28436181

[366] Vucic S Cheah BC Yiannikas C Kiernan MC. Cortical excitability distinguishes ALS from mimic disorders. Clin Neurophysiol. (2011) 122:1860–6. 10.1016/j.clinph.2010.12.062 21382747

[367] Floyd AG Yu QP Piboolnurak P Tang MX Fang Y Smith WA. Transcranial magnetic stimulation in ALS: utility of central motor conduction tests. Neurology. (2009) 72:498–504. 10.1212/01.wnl.0000341933.97883.a4 19204259 PMC2677511

[368] Mills KR. The natural history of central motor abnormalities in amyotrophic lateral sclerosis. Brain. (2003) 126(Pt. 11):2558–66. 10.1093/brain/awg260 12937082

[369] Vucic S Lin CS Cheah BC Murray J Menon P Krishnan AV. Riluzole exerts central and peripheral modulating effects in amyotrophic lateral sclerosis. Brain. (2013) 136(Pt. 5):1361–70. 10.1093/brain/awt085 23616585

[370] Feng Z Yasui Y. Statistical considerations in combining biomarkers for disease classification. Dis Mark. (2004) 20:45–51. 10.1155/2004/214152 15322313 PMC3839327

[371] Kuffner R Zach N Norel R Hawe J Schoenfeld D Wang L. Crowdsourced analysis of clinical trial data to predict amyotrophic lateral sclerosis progression. Nat Biotechnol. (2015) 33:51–7. 10.1038/nbt.3051 25362243

[372] Gomeni R Fava M. Amyotrophic lateral sclerosis disease progression model. Amyotroph Lateral Scler Frontotemporal Degener. (2014) 15:119–29. 10.3109/21678421.2013.838970 24070404

[373] Lunetta C Lizio A Melazzini MG Maestri E Sansone VA. Amyotrophic Lateral Sclerosis Survival Score (ALS-SS): a simple scoring system for early prediction of patient survival. Amyotroph Lateral Scler Frontotemporal Degener. (2015) 17:93–100. 10.3109/21678421.2015.1083585 26470943

[374] Benatar M Boylan K Jeromin A Rutkove SB Berry J Atassi N. ALS biomarkers for therapy development: state of the field and future directions. Muscle Nerve. (2016) 53:169–82. 10.1002/mus.24979 26574709 PMC4718795

